# Genotranscriptomic meta‐analysis of the CHD family chromatin remodelers in human cancers – initial evidence of an oncogenic role for CHD7

**DOI:** 10.1002/1878-0261.12104

**Published:** 2017-07-21

**Authors:** Xiaofang Chu, Xuhui Guo, Yuanyuan Jiang, Huimei Yu, Lanxin Liu, Wenqi Shan, Zeng‐Quan Yang

**Affiliations:** ^1^ Department of Oncology Wayne State University School of Medicine Detroit MI USA; ^2^ Department of Breast Surgery Affiliated Cancer Hospital of Zhengzhou University Henan China; ^3^ College of Basic Medicine Jilin University Changchun China; ^4^ Molecular Therapeutics Program Barbara Ann Karmanos Cancer Institute Detroit MI USA

**Keywords:** breast cancer, chromatin remodeler, chromodomain helicase DNA binding protein, copy number alteration

## Abstract

Chromodomain helicase DNA binding proteins (CHDs) are characterized by N‐terminal tandem chromodomains and a central adenosine triphosphate‐dependent helicase domain. CHDs govern the cellular machinery's access to DNA, thereby playing critical roles in various cellular processes including transcription, proliferation, and DNA damage repair. Accumulating evidence demonstrates that mutation and dysregulation of CHDs are implicated in the pathogenesis of developmental disorders and cancer. However, we know little about genomic and transcriptomic alterations and the clinical significance of most CHDs in human cancer. We used TCGA and METABRIC datasets to perform integrated genomic and transcriptomic analyses of nine CHD genes in more than 10 000 primary cancer specimens from 32 tumor types, focusing on breast cancers. We identified associations among recurrent copy number alteration, gene expression, clinicopathological features, and patient survival. We found that *CHD7* was the most commonly gained/amplified and mutated, whereas *CHD3* was the most deleted across the majority of tumor types, including breast cancer. Overexpression of *CHD7* was more prevalent in aggressive subtypes of breast cancer and was significantly correlated with high tumor grade and poor prognosis. CHD7 is required to maintain open, accessible chromatin, thus providing fine‐tuning of transcriptional regulation of certain classes of genes. We found that CHD7 expression was positively correlated with a small subset of classical oncogenes, notably *NRAS*, in breast cancer. Knockdown of CHD7 inhibits cell proliferation and decreases gene expression of several CHD7 targets, including *NRAS*, in breast cancer cell lines. Thus, our results demonstrate the oncogenic potential of CHD7 and its association with poor prognostic parameters in human cancer.

**Abbreviations**
AJCC: The American Joint Committee on Cancer
ATP: adenosine triphosphate
CAN: copy number alteration
CDK8: cyclin‐dependent kinase 8
CHD: chromodomain helicase DNA binding
ChIP: chromatin immunoprecipitation
CR: cysteine‐rich
INO80: inositol requiring 80
ISWI: imitation SWI
METABRIC: Molecular Taxonomy of Breast Cancer International Consortium
MYCN: v‐Myc avian myelocytomatosis viral oncogene neuroblastoma‐derived homolog
NPI: Nottingham prognostic index
NRAS: neuroblastoma RAS viral oncogene homolog
NuRD: nucleosome remodeling histone deacetylase
SANT: switching‐defective protein 3, adaptor 2, nuclear receptor corepressor, transcription factor IIIB
SEC63: SEC63 homolog, protein translocation regulator
SOX2: SRY‐box 2
SRC: SRC proto‐oncogene, nonreceptor tyrosine
SWI/SNF: switching‐defective/sucrose non‐fermentable
TCGA: the cancer genome atlas

## 1 Introduction

Adenosine triphosphate (ATP)‐dependent chromatin remodelers govern the cellular machinery's access to DNA. Hence, they play critical roles in various cellular processes, including transcription, proliferation, and DNA damage repair (Clapier and Cairns, 2009; Murawska and Brehm, 2011). Eukaryotic chromatin remodelers are divided into the following four families according to their protein similarity and domain structure: switching‐defective/sucrose non‐fermentable (SWI/SNF), imitation SWI (ISWI), inositol requiring 80 (INO80), and chromodomain helicase DNA binding (CHD). The CHD family, which consists of nine members (CHD1–CHD9), is characterized by two consecutive chromodomains in the N‐terminal region and an ATPase domain in the central region (Barrett *et al*., 2007). Based on other significant structural motifs and functional complexes, CHD proteins are divided into three subfamilies (Alhazzazi *et al*., 2011). CHD1 and CHD2 are class I CHD proteins, which are distinguished by a C‐terminal DNA‐binding domain with a preference for binding AT‐rich DNA sequences (Delmas *et al*., 1993; Stokes and Perry, 1995). CHD3, CHD4, and CHD5 are class II CHD proteins, which are distinguished by a pair of N‐terminal plant homeodomain zinc finger domains and a lack of DNA‐binding domain (Schuster and Stoger, 2002). CHD6, CHD7, CHD8, and CHD9 are class III CHD proteins, which are distinguished by a C‐terminal duplicated Brahma and Kismet (BRK) domains, a switching‐defective protein 3, adaptor 2, nuclear receptor corepressor, transcription factor IIIB (SANT) domain, cysteine‐rich (CR) domain, and a DNA‐binding domain (Chiba *et al*., 1994; Schuster and Stoger, 2002; Shur and Benayahu, 2005). Regardless of overall protein domain structure, the function of CHD superfamily proteins is intimately tied to regulating gene expression by modulating chromatins.

Dysregulation of CHD chromatin remodelers is a pivotal event in various human diseases, notably cancer and developmental disorders (Li and Mills, 2014; Mills, 2017). For example, *CHD1* is one of the most frequently deleted genes in prostate cancer. A recent study demonstrated that loss of CHD1 causes DNA repair defects and has the potential to enhance prostate cancer therapeutic responsiveness (Kari *et al*., 2016). Class II CHD proteins are the catalytic components of the nucleosome remodeling histone deacetylase (NuRD) complex (Stanley *et al*., 2013). CHD4 deficiency was shown to reduce the recruitment of homologous recombination repair factor BRCA1, and it impaired the efficiency of homologous recombination repair, which could affect the treatment of breast cancer characterized by BRCA1/BRCA2 mutations (Abdelmohsen *et al*., 2012). Using high‐throughput genomics, Geeleher *et al*. (2015) revealed that expression of CHD4 predicted the sensitivity of the histone deacetylase inhibitor vorinostat in a large panel of cancer cell lines. We previously reported that CHD3 and CHD4 are commonly mutated in a subset of breast cancers (Yu *et al*., 2017). Furthermore, a recent study revealed that CHD4 has an oncogenic role in maintaining epigenetic silencing of tumor suppressor genes in colorectal cancer (Xia *et al*., 2017). CHD7 expression predicts survival outcomes in patients with resected pancreatic cancer (Colbert *et al*., 2014). Furthermore, loss‐of‐function mutations in several CHD genes are associated with developmental disorders and intellectual disability (Li and Mills, 2014). Notably, *de novo* mutations in CHD7 cause the CHARGE syndrome (coloboma, heart defects, atresia of the choanae, retarded growth and development, genitourinary hypoplasia, and ear abnormalities, including deafness and vestibular disorders), which is characterized by a unique combination of organ anomalies (Basson and van Ravenswaaij‐Arts, 2015).

Previous studies revealed the importance of several CHDs in cancer pathogenesis and therapeutic responsiveness (Geeleher *et al*., 2015; Kadoch and Crabtree, 2015; Li and Mills, 2014). However, our knowledge of the genomic and transcriptomic alterations of CHD genes and the clinical significance of those alterations in human cancer remains incomplete. In the present study, we performed a genotranscriptomic meta‐analysis of nine CHDs in more than 10 000 cancer samples across 32 tumor types. We identified the frequency of copy number alteration (CNA), mutations, and aberrant expression for each CHD in a broad spectrum of human cancers. We then focused on human breast cancer, one of the most common cancers, resulting in more than 450 000 deaths each year worldwide. We investigated the associations between recurrent CNA and gene expression level of each CHD, clinicopathological features, overall survival, and disease‐free survival of patients with breast cancer. Our studies demonstrate the oncogenic potential of CHD7 and prioritize a subset of CHDs for future research focused on understanding molecular mechanisms and therapeutic potentials.

## 2 Materials and methods

### 2.1 Genomic and clinical data on TCGA and METABRIC cancer samples

Genetic and expression alteration data from 11 313 tumor samples spanning 32 tumor types in provisional the cancer genome atlas (TCGA) studies were obtained from the cBio Cancer Genomics Portal (http://www.cbioportal.org) (Cerami *et al*., 2012; Gao *et al*., 2013; Jiang *et al*., 2016; Liu *et al*., 2016a). Integrative analysis of cancer genomics and clinical data has been described in detail earlier (Jiang *et al*., 2016; Liu *et al*., 2016a). In the cBio portal, the copy number for each CHD gene was generated by the genomic identification of significant targets in cancer (GISTIC) algorithm and categorized as copy number level per gene: ‘−2’ is a deep loss (possibly a homozygous deletion), ‘−1’ is a heterozygous deletion, ‘0’ is diploid, ‘1’ indicates a low‐level gain, and ‘2’ is a high‐level amplification. For mRNA expression data, the relative expression of an individual gene and the gene's expression distribution in a reference population were analyzed. The reference population consisted of tumors that are diploid for the gene in question. The returned value indicates the number of standard deviations from the mean of expression in the reference population (*Z*‐score). Somatic mutation data were obtained by exome sequencing (Cerami *et al*., 2012; Gao *et al*., 2013). Breast cancer subtype and clinicopathologic information were obtained from a previous publication and extracted via the UCSC Cancer Genomics Browser (genome‐cancer.ucsc.edu) and the cBio Cancer Genomics Portal (Cancer Genome Atlas Network, 2012; Cerami *et al*., 2012). Of the 960 breast cancer samples, 808 had subtype data available, namely 22 normal‐like, 405 luminal A, 185 luminal B, 66 HER2+, and 130 basal‐like breast cancers (Cerami *et al*., 2012; Liu *et al*., 2016a). The Molecular Taxonomy of Breast Cancer International Consortium (METABRIC) dataset contains ~ 2000 primary breast cancers with long‐term clinical follow‐up data. A detailed description of the dataset is presented in the original publication (Curtis *et al*., 2012). The CNAs and normalized expression data from the METABRIC database were downloaded with access permissions from the European Genome‐phenome Archive (https://www.ebi.ac.uk/ega) under accession number EGAC00000000005 as well as from the cBio Cancer Genomics Portal (Cerami *et al*., 2012). In the METABRIC dataset, 1974 samples had subtype data available, namely 199 normal‐like, 718 luminal A, 488 luminal B, 240 HER2+, and 329 basal‐like breast cancers (Curtis *et al*., 2012).

### 2.2 Semiquantitative PCRs

To assess gene expression at the mRNA level, RNA was prepared from human breast cancer cell lines and the MCF10A cell line by using an RNeasy Plus Mini Kit (Qiagen, Germantown, MD, USA) (Jiang *et al*., 2016). RNA was mixed with qScript cDNA SuperMix (Quanta Biosciences, Gaithersburg, MD, USA) and then converted to cDNA through a reverse transcription (RT) reaction for real‐time PCRs. Primer sets were obtained from Life Technologies (Carlsbad, CA, USA). A PUM1 primer set was used as a control. Semiquantitative RT‐PCR was performed using the FastStart Universal SYBR Green Master (Roche Diagnostics, Indianapolis, IN, USA) as described earlier (Jiang *et al*., 2016; Liu *et al*., 2016a).

### 2.3 Cell culture and growth assays

The SUM cell lines were obtained from Stephen P. Ethier, and all other cell lines in this study were obtained from American Type Culture Collection (Manassas, VA, USA). Our cultures for the SUM breast cancer cell lines and an immortalized, nontransformed human mammary epithelial MCF10A cell line have been described in detail (Liu *et al*., 2009; Yang *et al*., 2006). All cell lines were tested routinely and authenticated using cell morphology, proliferation rate, a panel of genetic markers, and contamination checks. To determine the contribution of endogenous CHD7 overexpression on the growth of human breast cancer *in vitro*, in the HCC1187 and SUM102 breast cancer cell lines, we knocked down CHD7 with small interfering RNA (siRNA). siRNA were purchased from Sigma Aldrich (St. Louis, MO, USA). As a negative control, we used a MISSION siRNA Universal Negative Control. For transfection, cells were seeded in appropriate cell culture plates and maintained overnight under standard conditions. Plate sizes, cell densities, and siRNA quantities depended on the cell line and the experimental setup; 10–30 nm siRNA was transfected using the MISSION siRNA transfection reagent according to the manufacturer's protocol (Sigma Aldrich). Five days after siRNA transfection, 3‐(4,5‐dimethylthiazol‐2‐yl)‐2,5‐diphenyltetrazolium bromide (MTT) assays were performed as described earlier (Jiang *et al*., 2016; Liu *et al*., 2016a).

### 2.4 Immunoblotting and antibodies

Immunoblot assays were performed as previously described (Jiang *et al*., 2016; Liu *et al*., 2016a). Briefly, whole‐cell lysates were prepared by scraping cells from the dishes into cold RIPA lysis buffer. After centrifugation, protein content was estimated by the Bradford method. A total of 20–50 μg of total cell lysate was resolved by sodium dodecyl sulfate/polyacrylamide gel electrophoresis and transferred onto a polyvinylidene difluoride membrane. Antibodies used in the study included anti‐CHD7 (1 : 1000; Bethyl Laboratories A300‐705A‐T, Montgomery, TX, USA) and anti‐β‐actin (1 : 5000; Sigma Aldrich A5441).

### 2.5 Statistical analysis

Statistical analyses were performed using r software (http://www.r-project.org) and graphpad Prism (version 6.03; GraphPad Software, Inc., La Jolla, CA, USA) (Jiang *et al*., 2016; Liu *et al*., 2016a). The significance of the difference in mRNA expression level for each CHD among different subtypes, stages, and grades of breast cancer samples was calculated using ANOVA and Welch's *t*‐test as described earlier (Jiang *et al*., 2016; Liu *et al*., 2016a). To analyze the relationships between CHD mRNA expression and overall patient survival in breast cancer, samples were divided into high and low expression groups for each CHD, based on mRNA expression *Z*‐scores in TCGA and METABRIC cancer samples.

## 3 Results

### 3.1 Copy number alternations of CHD genes in human cancers

**Figure 1:**
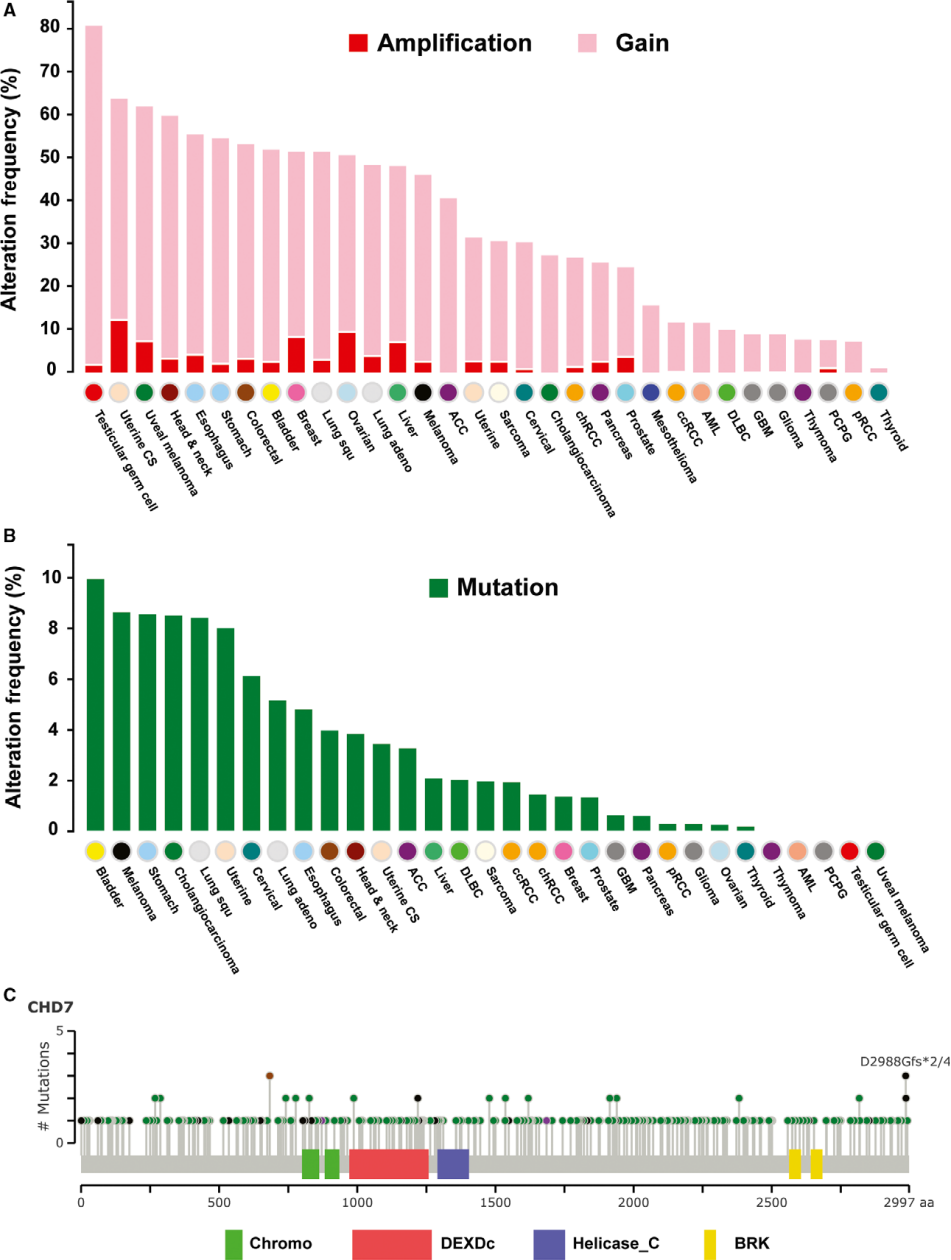
Genetic alterations of CHD7 in a spectrum of human cancers. (A) Frequencies of CHD7 gain and amplification across 32 TCGA tumor types. (B) Frequencies of CHD7 somatic mutation across 31 TCGA tumor types (excluding mesothelioma, as its mutation data were not available). (C) Mutational spectra of CHD7 gene in human tumors. The images show protein domains and the positions of CHD7 somatic mutations in 31 TCGA tumor types. A green dot indicates a missense mutation, a black dot indicates a truncated mutation, a brown dot indicates an in‐frame insertion or deletion, and a pink dot indicates other mutation. The data were obtained from TCGA database via cBioPortal.

As the first step in our systematic meta‐analysis to determine the spectrum of genetic alterations in CHD genes in human cancers, we queried CNAs of nine CHD genes compiled from 11 313 tumor samples spanning 32 tissue types in TCGA via cBioPortal (Table S1). The copy number for each CHD was generated by the copy number analysis algorithm GISTIC and categorized according to copy number level per gene as high‐level amplification, low‐level gain, diploid, heterozygous deletion, and homozygous deletion. We combined the copy number grouping into amplification/gain (high‐level amplification and low‐level gain), diploid, and deletion (heterozygous or homozygous deletions). Among 10 845 TCGA samples with CNA data available, we found that *CHD6* and *CHD7* were amplified/gained and *CHD3* was deleted in more than 35% of patient samples. Among 32 tumor types, *CHD7* was more frequently (> 50%) amplified/gained in 11 tumor types, including breast, lung, colorectal, and ovarian cancers, and *CHD6* was more frequently (> 50%) amplified/gained in eight TCGA tumor types, whereas *CHD3* was more frequently deleted (> 50%) in nine tumor types (Fig. 1A and Table S2). Furthermore, among 10 845 TCGA tumor samples, *CHD7* had high‐level amplification in 362 cases (0.9%), in which five tumor types, namely breast, ovarian, uterine, and liver cancers and melanoma, exhibited *CHD7* amplification in more than 5% of cases (Fig. 1A and Table S2). In contrast, *CHD6* had high‐level amplification in 179 TCGA samples (0.47%), in which two types, colorectal and uterine cancers, exhibited *CHD6* amplification in more than 5% of samples. We also found homozygous deletions of *CHD1* and *CHD3* in 119 (0.01%) and 103 (0.01%) TCGA cases, respectively. Strikingly, prostate cancer had a dramatically higher frequency of homozygous deletions of *CHD1* and *CHD3*, in 10.2% and 6.7% of cases, respectively. In summary, among nine CHD genes, *CHD6* and *CHD7* had the highest frequency of genetic gain/amplification, whereas *CHD1* and *CHD3* were most commonly deleted in a spectrum of human cancers.

### 3.2 Mutation frequencies and spectra of CHD genes in human cancers

To investigate the mutation frequencies and spectra of CHD genes in human cancers, we analyzed somatic mutation profiles of CHD genes in TCGA dataset. In 7978 sequenced TCGA tumor samples, four CHD genes—CHD4, CHD5, CHD6, and CHD7—exhibited mutations in more than 200 tumor samples. We previously reported the mutation spectra for *CHD4* and *CHD5* in TCGA breast cancers (Yu *et al*., 2017). Here, we analyzed the mutation spectra of *CHD6* and *CHD7* genes in human cancers.

In TCGA samples, 233 of 7978 sequenced tumor samples contained the following 274 CHD6 mutations: 234 missense, one in‐frame insertion and one in‐frame deletion, 35 truncating, and three other mutations (Table S3). In 31 tumor types (excluding mesothelioma, as its mutation data were not available), we found that *CHD6* was mutated in more than 5% of five tumor types, namely uterine, stomach, bladder, colorectal cancers, lung adenocarcinoma, and melanoma. Melanoma had the most frequent mutations (41 of 368 melanoma samples, 11.1%; Table S3).

In TCGA samples, 236 sequenced tumor samples contained a total of 291 *CHD7* mutations: 250 missense, two in‐frame insertions, 37 truncating, and two other mutations (Table S4). We found that *CHD7* was mutated in more than 5% of eight tumor types, namely bladder, stomach, uterine, and cervical cancers, as well as cholangiocarcinoma, lung squamous cell carcinoma, lung adenocarcinoma, and melanoma (Fig. 1B). Among 31 tumor types, bladder cancer exhibited the most frequent *CHD7* mutations (13 of 130 sequenced tumor samples, 10%; Fig. 1B). Similar to *CHD7* mutations in the CHARGE syndrome, these mutations in human cancers were also distributed throughout the entire coding region of *CHD7* genes (Fig. 1C) (Basson and van Ravenswaaij‐Arts, 2015). However, in contrast to the most prevalent loss‐of‐function mutation of *CHD7*, such as nonsense mutation and frameshift deletion or insertion in CHARGE syndrome, the most frequent (86%) *CHD7* mutations in human cancers were missense mutations.

### 3.3 Molecular profiling of CHD7 genes in different subtypes of breast cancer

Breast cancer is the most common cancer and one of the leading causes of cancer death among women. Using gene expression profiling, breast cancer has been classified into five molecular subtypes: luminal A, luminal B, epidermal growth factor receptor 2‐enriched (HER2+), basal‐like, and normal‐like breast cancers (Cancer Genome Atlas Network, 2012; Perou *et al*., 2000; Riaz *et al*., 2013). Basal‐like (closely related to triple‐negative) breast cancer tends to occur in young women and presents with an aggressive course, recurrence, distant metastasis, and shorter survival (Bertucci *et al*., 2012; Burstein *et al*., 2015; Carey *et al*., 2006). In 960 TCGA breast cancers that have mRNA, CNA, and sequencing data, we found that the most commonly amplified/gained (> 50%) CHD gene was *CHD7*, and the most deleted (> 50%) CHD genes were *CHD3* and *CHD9*. Notably, *CHD7* was highly amplified in 8.85% but mutated in 1.25% and homozygously deleted in only 0.1% of TCGA breast cancers. In contrast, *CHD6* exhibited high‐level amplification in only 1.98% of TCGA breast cancers. Furthermore, *CHD7* was overexpressed (*Z*‐score ≥ 1) in 33.96% and *CHD3* was underexpressed (*Z*‐score ≤ −1) in 35.1% of TCGA breast cancers (Table 1).

**Table 1:** Frequency (%) of CHD genetic alterations and expression levels in 960 TCGA breast cancers

Gene	Location	DNA alterations						mRNA expression levels		
		Amp	Gain	Diploid	Hetloss	Homdel	Mutation	*Z* score ≥ 1	1 > *Z* score > −1	*Z* score ≤ −1
CHD1	5q15‐q21	0.21	18.13	55.83	25.00	0.83	0.63	8.44	89.79	1.77
CHD2	15q26	3.54	13.75	57.60	25.00	0.10	0.94	11.35	69.58	19.06
CHD3	17p13.1	0.10	5.42	33.44	**60.31**	0.73	1.46	6.25	58.65	**35.10**
CHD4	12p13	3.33	21.88	60.42	14.17	0.21	2.08	19.27	64.58	16.15
CHD5	1p36.31	0.73	6.56	53.23	**38.85**	0.63	1.46	1.67	98.33	0.00
CHD6	20q12	1.98	**40.10**	51.35	6.46	0.10	1.98	21.04	64.79	14.17
CHD7	8q12.2	**8.85**	**44.06**	41.25	5.73	0.10	1.25	**33.96**	55.31	10.73
CHD8	14q11.2	0.63	17.40	59.79	22.08	0.10	1.46	17.92	60.00	22.08
CHD9	16q12.2	1.88	12.60	29.38	**55.10**	1.04	1.46	9.58	71.88	18.54

Amp, high‐level amplification; Gain, low‐level gain; Hetloss, heterozygous deletion; Homdel, homozygous deletion. Numbers in bold indicate higher frequencies of DNA or mRNA alternations (Amp > 5%; Gain and Hetloss > 35%, and mRNA (Z score) up‐ or down‐regulation  >30%).

**Figure 2:**
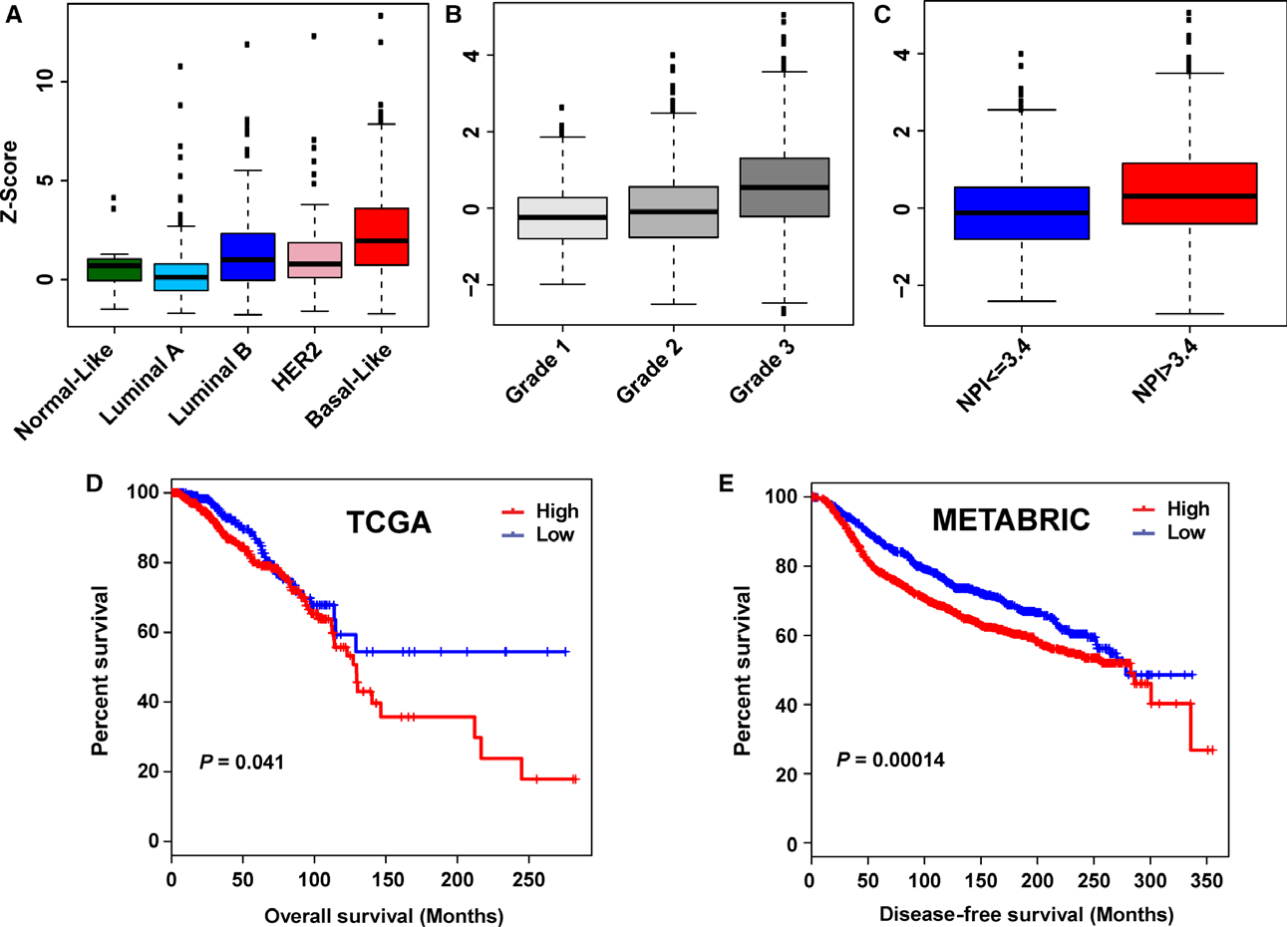
CHD7 expression is significantly associated with aggressiveness and poorer prognosis of breast cancer. (A) Expression levels of CHD7 across five subtypes of TCGA breast cancer samples. (B) CHD7 was significantly more highly expressed in Grade 3 compared with that in Grade 1 and Grade 2 METABRIC breast cancers (*P *<* *0.001). (C) Patients with a poor prognosis (NPI > 3.4) have significantly increased levels of CHD7 expressed in their tumors compared with those with a good prognosis (*P *<* *0.001). (D) Kaplan–Meier plots of overall survival associated with mRNA expression levels of CHD7 in TCGA breast cancers. (E) Kaplan–Meier plots of disease‐free survival associated with mRNA expression levels of CHD7 in METABRIC breast cancers.

To determine whether genetic alteration or mRNA expression of each CHD gene is specific to a breast cancer subtype, we analyzed CNA and mRNA expression across five breast cancer subtypes in TCGA cohort. The frequencies of copy number, somatic mutation, and expression level of CHD genes in five breast cancer subtypes are shown in Table S5. We found that *CHD7* was more commonly (> 50%) amplified/gained in aggressive luminal B, HER2+, and basal‐like subtypes (Table S5). We also found that *CHD7* was overexpressed (*Z*‐score ≥ 1) in more than 50% of luminal B and basal‐like breast cancers. Notably, among nine CHDs, CHD7 was overexpressed (*Z*‐score ≥ 1) in 71.54% of TCGA basal‐like breast cancers, compared to 20.49% of luminal A subtype (*P *< 0.01; Fig. 2A and Table S5).

To validate our findings from TCGA dataset regarding CHD genetic alterations in breast cancer, we conducted an independent analysis using the METABRIC breast cancer dataset, which contains ~ 2000 primary breast cancers with long‐term clinical follow‐up data. Here, too, *CHD7* was the most commonly amplified/gained CHD gene (Table S6), although the frequency of gain/amplification in the METABRIC dataset was lower than that of the TCGA dataset, possibly due to the different CNA analysis platforms. Also, mRNA expression levels of CHD7 were significantly higher in basal‐like breast cancers (*P *< 0.01; Fig. S1 and Table S7).

### 3.4 CHD7 expression is significantly associated with poorer prognosis of breast cancer

To investigate the clinicopathological relevance of CHDs in breast cancer, we next examined expression levels of each CHD gene at different stages [The American Joint Committee on Cancer (AJCC)] and histologic grades of breast cancers. CHD7 was dramatically more highly expressed in advanced stages and in higher grades of breast cancer (Fig. 2B). The Nottingham prognostic index (NPI), a clinicopathological classification system based on tumor size, histologic grade, and lymph node status that is widely used in Europe for breast cancer prognostication, was also available in the METABRIC cohort (Galea *et al*., 1992). Thus, we compared expression levels of nine CHDs between patients with high NPI (> 3.4) versus those with low NPI (≤ 3.4). As shown in Fig. 2C and Table S8, we found that among the nine CHD genes, only *CHD7* was significantly more highly expressed, while *CHD1*,* CHD6*, and *CHD9* were underexpressed in samples with high NPI. Next, we analyzed the relationship between CHD mRNA expression and overall and disease‐free survival of patients with breast cancer. We found that higher mRNA levels of CHD7 were significantly associated with shorter overall survival of TCGA breast cancer patients (*P *< 0.05; Fig. 2D). We validated that higher mRNA levels of CHD7 were also significantly associated with shorter overall survival of METABRIC breast cancer patients (*P *<* *0.001; Fig. S2). In the METABRIC dataset, disease‐free survival clinical information was available. We found that higher expression of CHD7 was significantly associated with shorter disease‐free survival (*P *<* *0.001) in METABRIC breast cancer patients (Fig. 2E).

### 3.5 CHD7 regulates a subset of cancer‐associated genes

**Figure 3:**
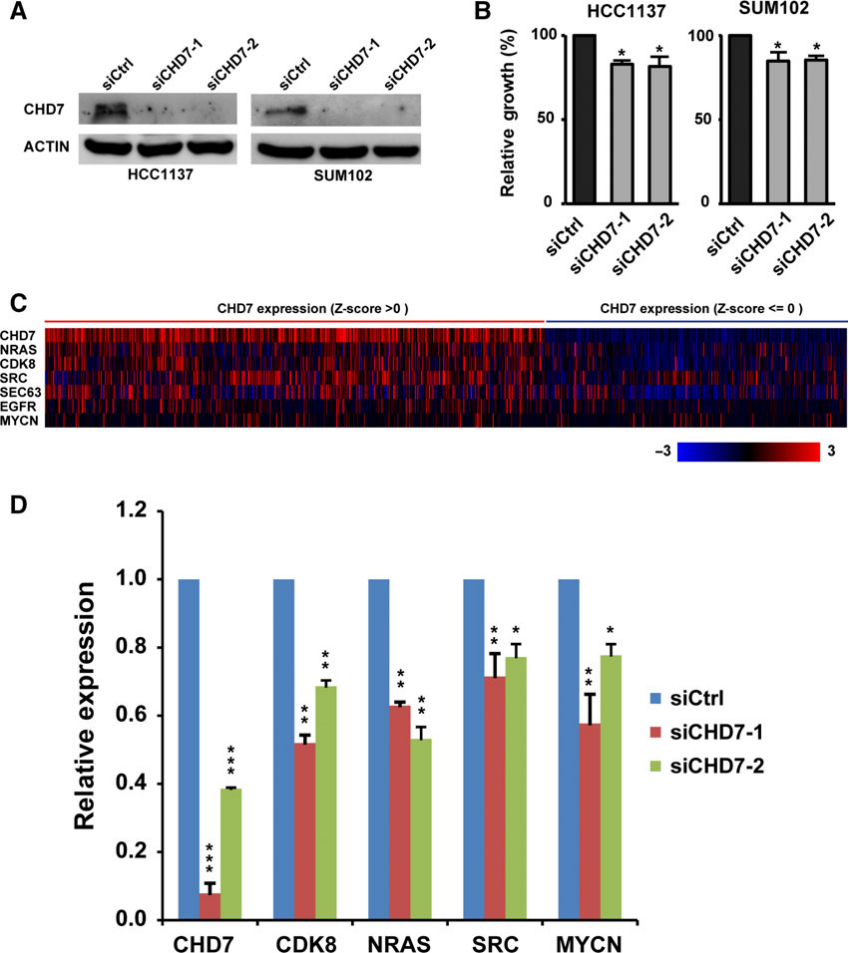
Knockdown of CHD7 inhibits cell proliferation and decreases gene expression of several CHD7 targets in breast cancer cell lines. (A) Knockdowns of CHD7 in HCC1187 and SUM102 cells with two different shRNA were confirmed by western blot assays. (B) Bar graph shows relative cell growth after knocking down CHD7 in HCC1187 and SUM102 breast cancer cells (*P  <  0.05). Data are expressed as mean ± SD. (C) mRNA expression heatmap of CHD7 and six CHD7 candidate target genes in 960 TCGA breast cancers. (D) qRT‐PCR assays show that CHD7 knockdown inhibited expression of four genes (*NRAS*,*CDK8*,*SRC*, and *MYCN*) in HCC1187 cells (*P <* *0.05, **P <* *0.01, and ***P <* *0.001, Student's *t*‐test).

Breast cancer cell lines and related animal models are essential tools with which to study cancer biology and to test novel therapeutic strategies. Thus, we next examined CHD7 expression in a panel of breast cancer cells. Figure S3 shows the expression level of CHD7 based on RNA sequencing data from 78 breast cancer cell lines compared with four normal mammary epithelial cell lines (Marcotte *et al*., 2016). Compared with MCF10A, an immortalized but nontumorigenic breast epithelial cell line, mRNA levels of CHD7 were more than twofold higher in 19 breast cancer cell lines, nine of them belonging to the basal subtype. We next performed qRT‐PCR assays and demonstrated that mRNA expression levels of CHD7 in HCC1187 and SUM102 breast cancer cell lines were more than twofold higher than that in MCF10A cells (data not shown). To assess the contribution of endogenous CHD7 overexpression on the transformation of human breast cancer, we examined the effects of knocking down CHD7 in HCC1187 and SUM102 cells. We obtained three siRNA targeting different regions of CHD7 genes. qRT‐PCR and western blot assays revealed that two siRNA decreased the expression of CHD7 at mRNA and protein levels (Fig. 3A). As shown in Fig. 3B, CHD7 knockdown slowed HCC1187 and SUM102 cell growth to ~ 70% of the growth of the nonsilenced control.

A previous study that used the approach of chromatin immunoprecipitation (ChIP) on tiled microarrays revealed that CHD7 targets active gene enhancer elements to modulate expression of specific sets of genes (Schnetz *et al*., 2009). Notably, CHD7 physically interacted with SRY‐box 2 (SOX2) transcriptional factor and regulated a set of common target genes associated with cancer and developmental disorders (Engelen *et al*., 2011). Using approaches of ChIP followed by massively parallel DNA sequencing and mRNA microarray, Engelen *et al*. identified 46 genes that are bound and activated by CHD7 and SOX2 in a neural stem cell model. To determine which of the 46 genes had higher positive or negative correlation with CHD7 expression in breast cancer, we analyzed the Spearman and Pearson correlations between expression levels of CHD7 and each CHD7‐SOX2‐targeted gene in TCGA breast cancer specimens from the cBioPortal database (Table S9). Higher weight was assigned to the Spearman correlation coefficient. We found that expression levels of four genes—cyclin‐dependent kinase 8 (*CDK8*), neuroblastoma RAS viral oncogene homolog (*NRAS*), SRC proto‐oncogene, nonreceptor tyrosine (*SRC*), and *SEC63* (SEC63 homolog, protein translocation regulator)—were positively correlated (Spearman's *r *> 0.3) with CHD7 expression (Fig. 3C). Furthermore, expression of the classical breast cancer oncogenes *EGFR* (epidermal growth factor receptor) and v‐Myc avian myelocytomatosis viral oncogene neuroblastoma‐derived homolog (*MYCN*) was also positively correlated with CHD7 expression, with Spearman's *r *= 0.24 and 0.12, respectively (Fig. 3C and Table S9). Next, we measured mRNA expression levels of six CHD7 candidate targets in HCC1187 cells after knocking down CHD7. We found that expression levels of four genes (*NRAS, CDK8, SRC*, and *MYCN*) decreased in CHD7‐knockdown HCC1187 cells (Fig. 3D). We also found that expression levels of *NRAS* and *MYCN*, but not others, decreased in CHD7‐knockdown SUM102 cells (Fig. S4). Thus, CHD7 likely modulates expression of a set of genes that are critical for cancer pathogenesis.

## 4 Discussion

In this study, we used a systematic genomics approach to assess the oncogenic properties of nine CHD genes across human tumor types. We found that *CHD6* and *CHD7* were most commonly gained/amplified or mutated, whereas *CHD1* and *CHD3* were most deleted in a spectrum of human cancers. Integrated genomic, transcriptomic, clinicopathological data, and *in vitro* siRNA‐mediated knockdown assays revealed the oncogenic potential of CHD7 in multiple cancer types, notably those arising from breast.

All CHD proteins are characterized by two consecutive chromodomains in the N‐terminal region. The chromodomain belongs to a larger, structurally related family of protein domains called Royal family domains, which include Tudor, malignant brain tumor (MBT), pro‐trp‐trp‐pro (PWWP), and Agenet domains. Based on the ChromoHub database, the human genome encodes 29 proteins that contain a chromodomain (Fig. S5), including nine CHDs and eight chromobox proteins (CBX 1–8). Notably, all nine CHD proteins have double chromodomains, but all other chromodomain‐containing proteins have only a single chromodomain. In general, the chromodomain binds methyl marks on histones with high specificity; such binding is coordinated by a hydrophobic cage formed by two, three, or four well‐conserved aromatic residues (Yap and Zhou, 2011). For example, the chromodomain is responsible for the direct interaction between heterochromatin protein 1 and trimethylated H3K9. Human CHD1 recognizes the di‐ and trimethylation of H3K4 through its two chromodomains and induces gene transcription. The chromodomains of CHD7 have a unique specificity for the monomethylated H3K4 mark, and recent studies have shown that CHD7 tracks H3K4 monomethylation patterns at enhancer motifs (Bajpai *et al*., 2010; Schnetz *et al*., 2009). Furthermore, CHD proteins contain additional epigenetic effector domains, including the plant homeodomain in class II family and SANT domain in class III family domains. Thus, chromodomains, together with other epigenetic recognition domains, likely play regulatory roles in recruiting CHD proteins to specific chromatin regions.

Chromodomain helicase DNA binding proteins are highly conserved from yeast to humans and have essential roles in controlling fundamental cellular processes of development (Hota and Bruneau, 2016). For example, CHD1 is essential during preimplantation embryonic development. The CHD3/4‐containing NuRD complex is involved in synapse formation and heart development (Garnatz *et al*., 2014; Yamada *et al*., 2014). CHD5 has critical roles during spermatogenesis (Govin *et al*., 2004). CHD7 is involved in a variety of stem cell differentiation and cell‐fate decisions. *De novo* mutations of CHD genes can lead to severe developmental disorders, including CHARGE syndrome, autism, and intellectual disability. Notably, *de novo* heterozygous mutations of the *CHD7* gene are the primary cause of the CHARGE syndrome (Schnetz *et al*., 2009). Most *CHD7* mutations in patients with CHARGE syndrome are nonsense mutations or frameshift deletions; missense mutations of *CHD7* are associated with milder CHARGE symptoms. The conditional deletion of *chd7* in mice recapitulates most symptoms of CHARGE syndrome (Sperry *et al*., 2014). Most *CHD7* mutations found in CHARGE syndrome affect ATP‐dependent chromatin remodeling (Basson and van Ravenswaaij‐Arts, 2015). A mutation in the first chromodomain (S834F) associated with CHARGE syndrome completely suppressed CHD7's remodeling activity (Bouazoune and Kingston, 2012). In this study, we revealed that in human cancer most *CHD7* mutations are missense, and those mutations were distributed throughout the entire coding region of the *CHD7* gene. We found that, of 291 *CHD7* mutations in TCGA tumors, 11 mutations were located at chromodomains and 25 mutations were at the ATP‐dependent helicase domain. Much work is needed to decipher the biological impacts and underlying mechanisms of these *CHD7* mutations on cancer pathogenesis.

Among nine CHDs, *CHD1* and *CHD3* were most commonly deleted in a spectrum of human cancers. Strikingly, homozygous deletions of *CHD1* and *CHD3* were found in 10.2% and 6.7% of prostate cancers, respectively. These findings agree with and consolidate prior reports on the genetic alterations and tumor‐suppressive functions of CHD1 and CHD3 in human cancer. Previous studies revealed that homozygous deletion of *CHD1* is the second most common genetic event in prostate cancer after *PTEN* deletion (Liu *et al*., 2012). Inactivation of *CHD1* is correlated with anchorage‐independent growth (Yu *et al*., 2015) and enhances the invasiveness of prostate cancer (Liu *et al*., 2012; Rodrigues *et al*., 2015). In breast cancer, the most commonly deleted/underexpressed CHD gene was *CHD3*. *CHD3* is localized to 17p13.1, the TP53 region. A recent study demonstrated that deletions linked to TP53 loss drive cancer through p53‐independent mechanisms (Liu *et al*., 2016b). In that study, a shRNA library targeting the ~ 100 protein‐coding genes (excluding *TP53*) in mouse chromosome 11B3 (syntenic to human 17p13.1) was screened for tumor‐suppressive activity in mouse models. Among 17 identified genes, *CHD3* was considered a potential tumor suppressor (Liu *et al*., 2016b).

A notable finding from our study is the dysregulation of CHD7 in a subset of human cancers. *CHD7* was highly amplified in more than 5% of samples among 11 tumor types, including breast, lung, colorectal, and ovarian cancers. A previous study revealed that *CHD7* is genetically altered in response to tobacco smoke in small‐cell lung cancer; either it has an in‐frame duplication of exons 3–7, or it is expressed as a fusion with *Pvt1 oncogene* (PVT1) (Pleasance *et al*., 2010). Another study found that CHD7 is highly expressed in human gliomas (Ohta *et al*., 2016). Recently, Colbert *et al*. reported that CHD7 was dysregulated in over 90% of their pancreatic ductal adenocarcinoma samples. Low CHD7 expression was associated with higher recurrence‐free survival and overall survival in patients receiving adjuvant gemcitabine (Colbert *et al*., 2014).

Studies of CHD7 in the CHARGE syndrome not only highlight the critical role of CHD7 in development but also indicate that CHD7 modulates central pathways in tumorigenesis. For example, CHD7 could be recruited to the p53 promoter and repress the expression of p53. Thus, loss of CHD7 contributed to the inappropriate activation of p53 and promoted the CHARGE phenotypes (Van Nostrand and Attardi, 2014). Another interesting study showed that CHD7 cooperates with SOX2 to regulate a small set of genes, such as NOTCH and Sonic Hedgehog pathway genes and classical oncogenes *NRAS* and *SRC*; these genes play critical roles in stem cell development and tumorigenesis (Engelen *et al*., 2011; Puc and Rosenfeld, 2011). SOX2 also plays a key role in the stem‐like cancer phenotype, particularly in squamous cell carcinoma (Ferone *et al*., 2016). Notably, a multiplatform analysis of 12 cancer types revealed that basal‐like breast cancer shares similar molecular features with squamous cell carcinoma. We also found that CHD7 and SOX2 (3q26) were likely cogained/amplified (*P *<* *0.0001) in breast cancer. In this study, we revealed that CHD7 likely regulates a small set of oncogenes, such as *NRAS* and *MYCN*, in breast cancer. We speculate that CHD7 allows transcription factors and the general transcription machinery access to DNA, thus promoting activation of certain classes of oncogenes in a cell type‐specific manner, which subsequently contributes to tumorigenesis.

## 5 Conclusion

We conducted a large‐scale genomic analysis of nine CHDs in human cancer, focusing on breast cancer. We found that *CHD6* and *CHD7* were the most commonly gained/amplified or mutated, whereas *CHD1* and *CHD3* were the most deleted CHDs in a spectrum of human cancers. Integrated genomic, transcriptomic, and clinicopathological data in ~ 3000 primary breast cancers revealed that different subtypes of breast cancer had distinctive copy number and expression patterns for each CHD. *CHD7* was the most upregulated CHD gene in breast cancer and was significantly associated with aggressiveness and poor prognosis of patients. Knockdown of *CHD7* inhibited cell proliferation in breast cancer cell lines. We found that CHD7 expression was positively correlated with a small subset of classical oncogenes, notably *NRAS* and *MYCN*, and validated that *CHD7* knockdown downregulated expression of them. Our findings provide a strong foundation for further mechanistic research and for developing therapies that target CHD7 or other CHDs in human cancer.

## Author contributions

XC, XG, YJ, and ZY designed research studies, analyzed data, and wrote manuscript. XC, XG, YJ, HY, LL, and WS conducted experiments and analyzed data. LL and ZY edited the manuscript.

## Supporting information


**Fig. S1**. Expression levels of CHD7 across five subtypes of METABRIC breast cancer samples.

**Fig. S2**. Kaplan‐Meier plots of overall survival associated with mRNA expression levels of CHD7 in METABRIC breast cancers.

**Fig. S3**. Expression levels of CHD7 based on RNA sequencing data from 78 breast cancer cell lines compared with four normal mammary epithelial cell lines.

**Fig. S4**. Expression levels of NRAS and MYCN, but not others, decreased in CHD7‐knockdown SUM102 cells (*P <* *0.05 and ***P <* *0.001, Student's *t*‐test).

**Fig. S5**. Phylogenetic analysis of chromodomain‐containing proteins. The image was obtained from the ChromoHub database (http://www.thesgc.org).

**Table S1**. The number of samples and data type in 32 TCGA databases.

**Table S2**. Frequency (%) of CHD genetic alterations in 32 tumor types from TCGA database.

**Table S3**. Mutations of CHD6 in TCGA tumors.

**Table S4**. Mutations of CHD7 in TCGATumors.

**Table S5**. Frequency (%) of CHD genetic alterations and expression levels in five subtypes of TCGA breast cancers.

**Table S6**. Frequency (%) of genetic and transcriptional alterations of CHDs in 1980 METABRIC breast cancers.

**Table S7**. Frequency (%) of CHD genetic alterations and expression levels in five subtypes of Metabric breast cancers.

**Table S8**. Expression levels of CHD genes associated with NPI score in METABRIC breast cancer.

**Table S9**. Correlation between mRNA expression of CHD7 and 46 candidate target genes in TCGA breast cancers.
