# Supplementary figures and images for: Genotranscriptomic meta‐analysis of the CHD family chromatin remodelers in human cancers – initial evidence of an oncogenic role for CHD7
Source: Mol Oncol. 2017 Jul 21;11(10):1348–60. doi: 10.1002/1878-0261.12104 (PMC5623824; doi:10.1002/1878-0261.12104)

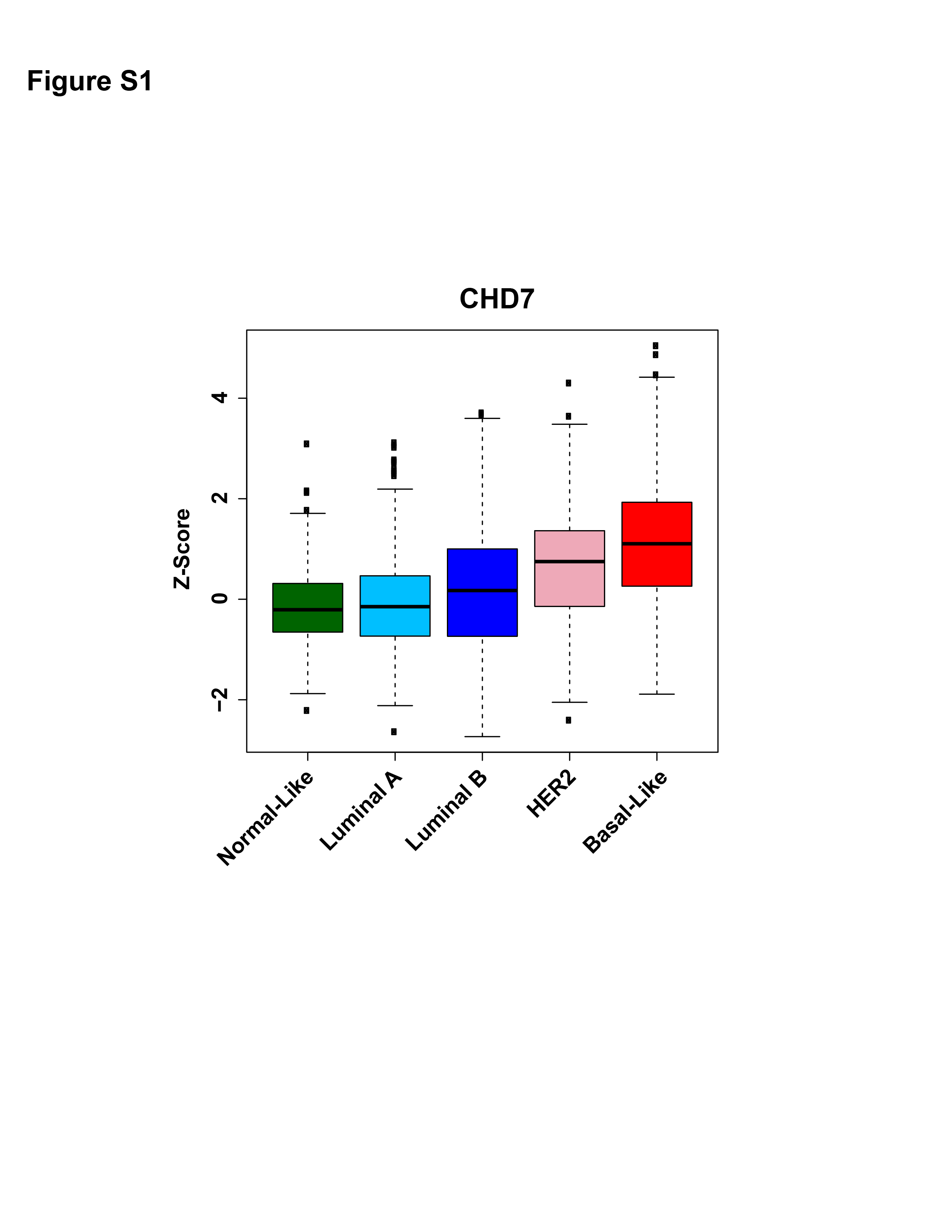

Supplement: Supplementary file 1 — Fig. S1. Expression levels of CHD7 across five subtypes of METABRIC breast cancer samples. [file MOL2-11-1348-s001.png]

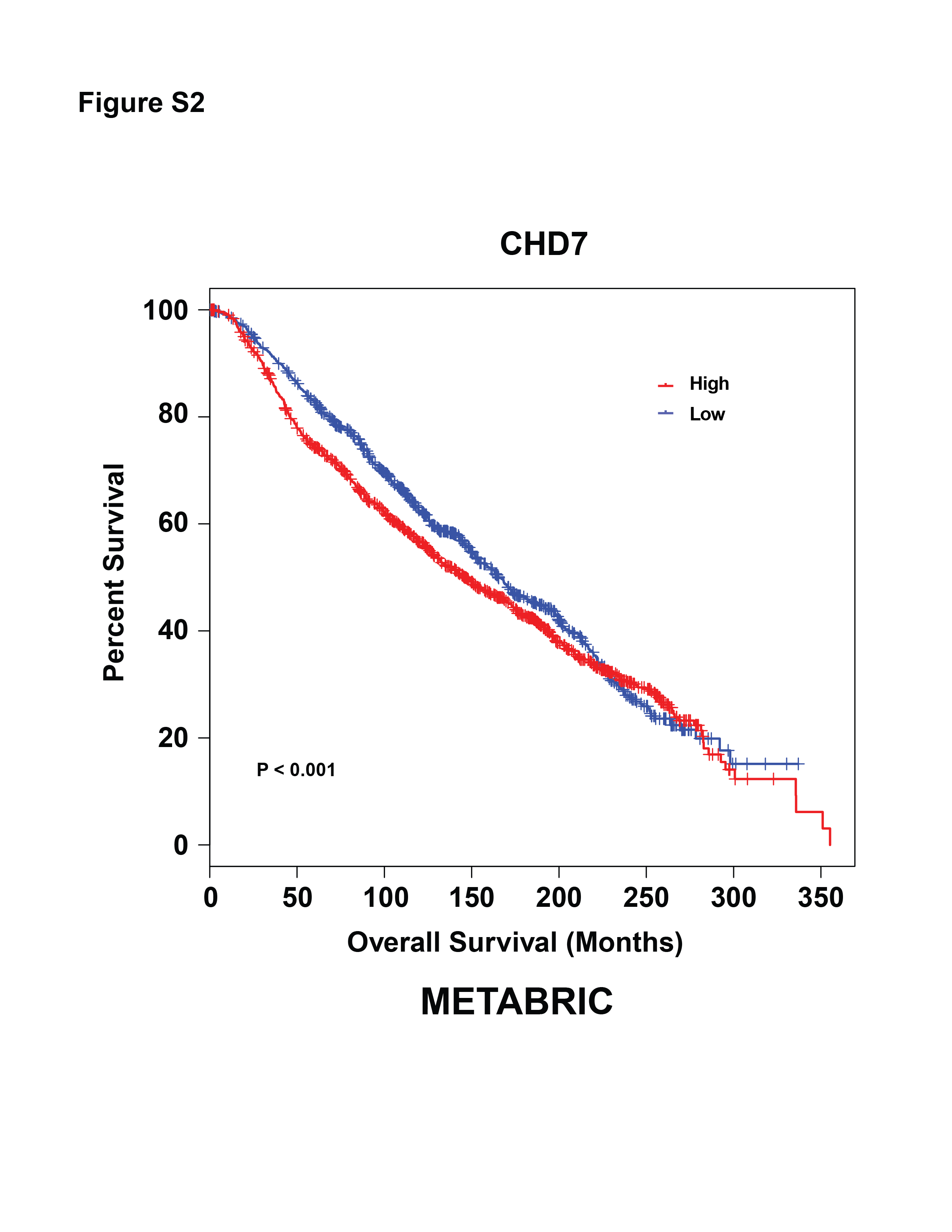

Supplement: Supplementary file 2 — Fig. S2. Kaplan‐Meier plots of overall survival associated with mRNA expression levels of CHD7 in METABRIC breast cancers. [file MOL2-11-1348-s002.png]

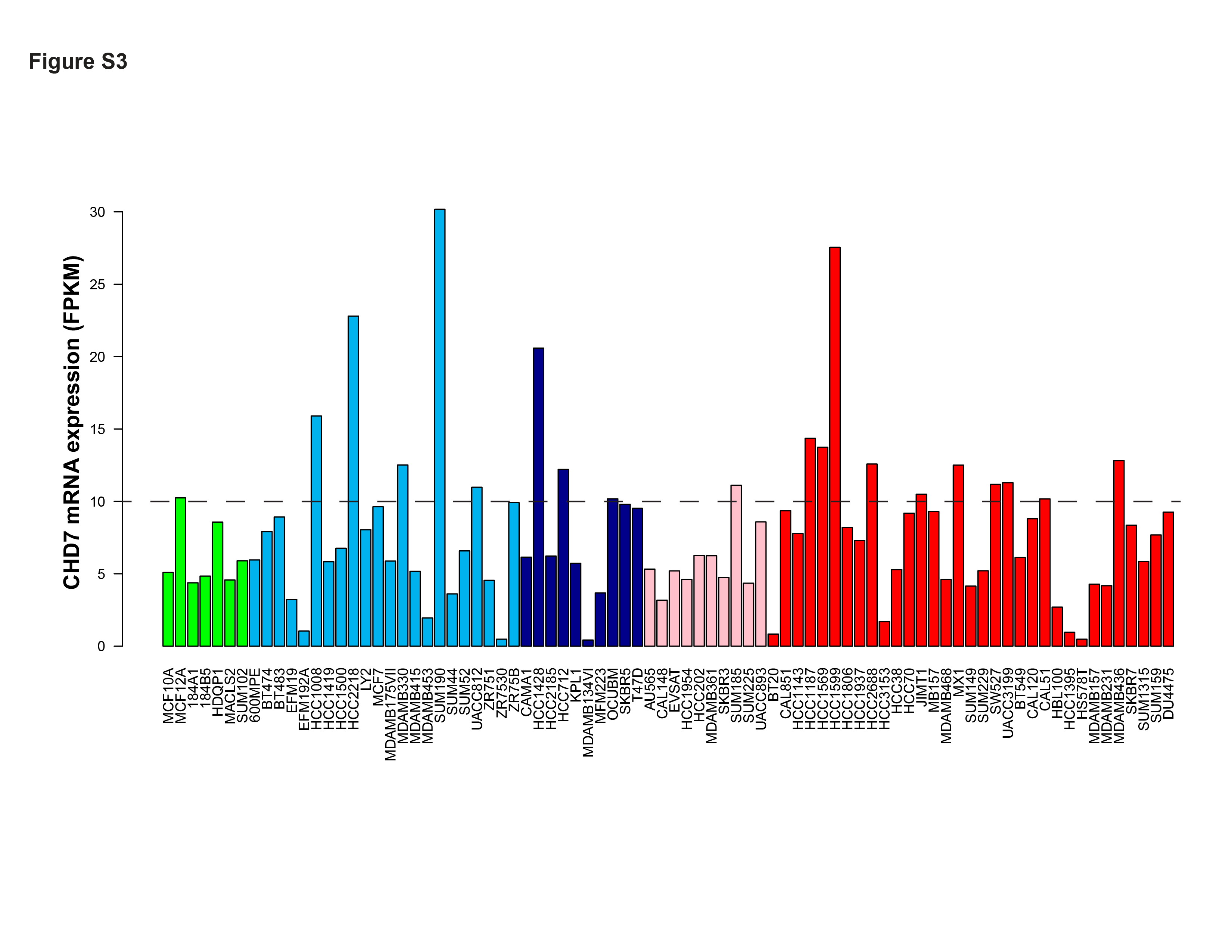

Supplement: Supplementary file 3 — Fig. S3. Expression levels of CHD7 based on RNA sequencing data from 78 breast cancer cell lines compared with four normal mammary epithelial cell lines. [file MOL2-11-1348-s003.png]

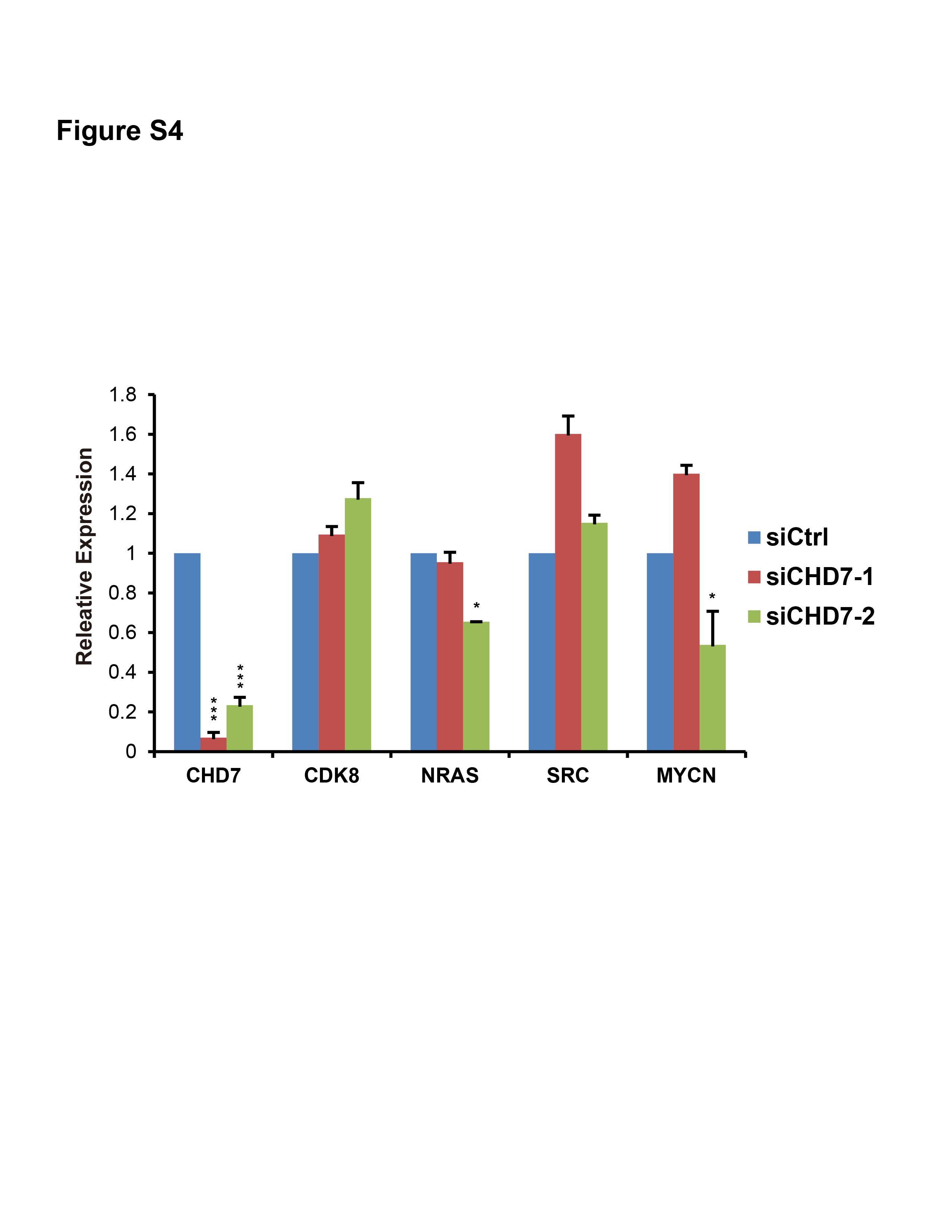

Supplement: Supplementary file 4 — Fig. S4. Expression levels of NRAS and MYCN, but not others, decreased in CHD7‐knockdown SUM102 cells (*P < 0.05 and ***P < 0.001, Student's t‐test). [file MOL2-11-1348-s004.png]

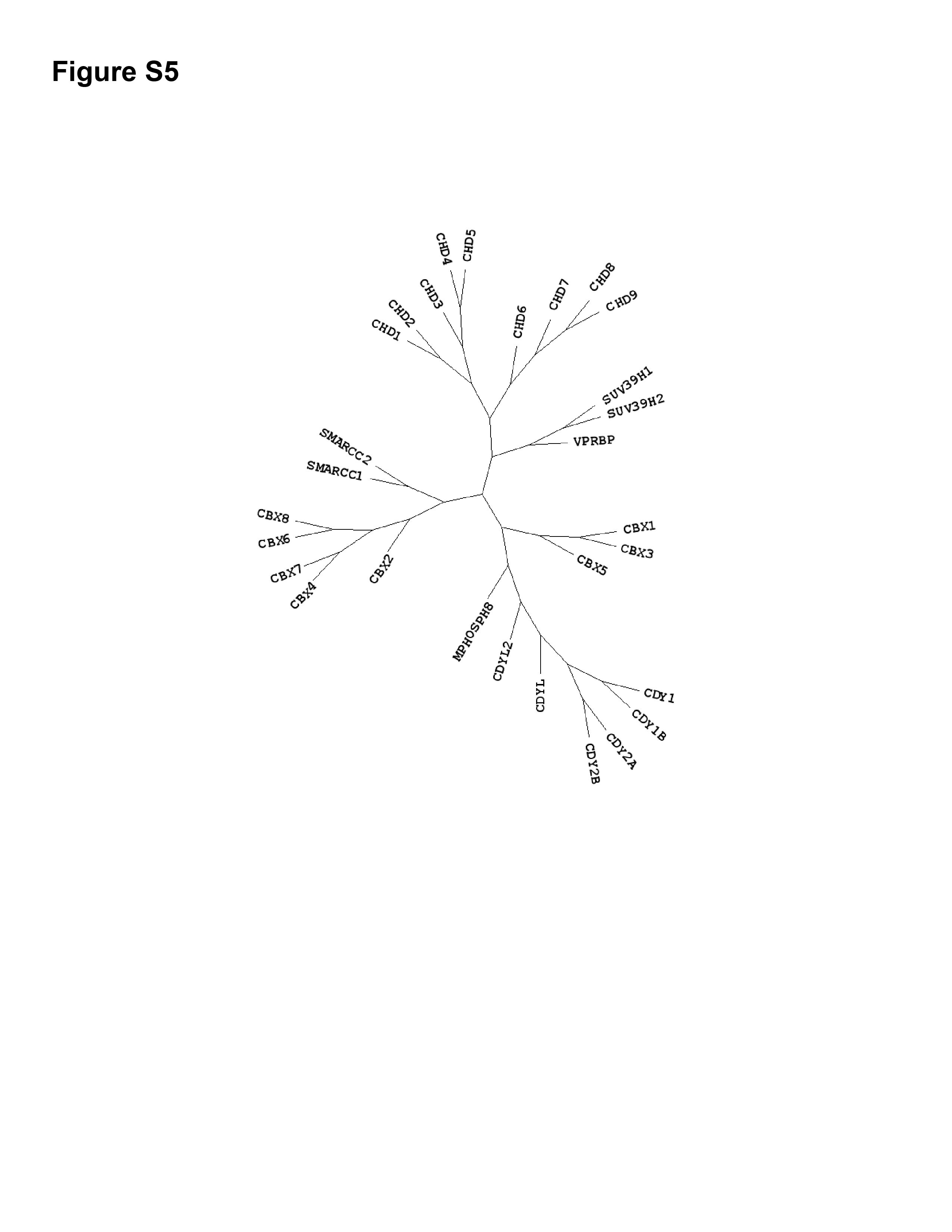

Supplement: Supplementary file 5 — Fig. S5. Phylogenetic analysis of chromodomain‐containing proteins. The image was obtained from the ChromoHub database (http://www.thesgc.org). [file MOL2-11-1348-s005.png]
