# Supplementary material for: Genotranscriptomic meta‐analysis of the CHD family chromatin remodelers in human cancers – initial evidence of an oncogenic role for CHD7
Source: Mol Oncol. 2017 Jul 21;11(10):1348–60. doi: 10.1002/1878-0261.12104 (PMC5623824; doi:10.1002/1878-0261.12104)
Supplement: Supplementary file 6 — Table S1. The number of samples and data type in 32 TCGA databases. [file MOL2-11-1348-s006.pdf]

Table S1. The number of samples and data type in 32 TCGA databases

Tumor Type	Description	Total	CNA	Tumor mRNA (RNA-Seq V2)	Sequenced
AML	Acute Myeloid Leukemia	200	191	173	197
ACC	Adrenocortical Carcinoma	92	90	79	90
Bladder	Bladder Urothelial Carcinoma	413	408	408	130
Glioma	Brain Lower Grade Glioma	530	513	530	286
Breast	Breast Invasive Carcinoma	1105	1080	1100	982
Cervical	Cervical Squamous Cell Carcinoma and Endocervical Adenocarcinoma	309	295	306	194
Cholangiocarcinoma	Cholangiocarcinoma	36	36	36	35
Colorectal	Colorectal Adenocarcinoma	633	616	382	223
Esophagus	Esophageal Carcinoma	186	184	185	185
GBM	Glioblastoma Multiforme	604	577	166	290
Head & neck	Head and Neck Squamous Cell Carcinoma	530	522	522	512
chRCC	Kidney Chromophobe	66	66	66	66
ccRCC	Kidney Renal Clear Cell Carcinoma	538	528	534	451
pRCC	Kidney Renal Papillary Cell Carcinoma	293	288	291	282
Liver	Liver Hepatocellular Carcinoma	442	370	373	373
Lung adeno	Lung Adenocarcinoma	522	516	517	230
Lung squ	Lung Squamous Cell Carcinoma	504	501	501	177
DLBC	Lymphoid Neoplasm Diffuse Large B-cell Lymphoma	48	48	48	48
Mesothelioma	Mesothelioma	87	87	87	N/A
Ovarian	Ovarian Serous Cystadenocarcinoma	603	579	307	316
Pancreas	Pancreatic Adenocarcinoma	186	184	179	150
PCPG	Pheochromocytoma and Paraganglioma	184	162	184	184
Prostate	Prostate Adenocarcinoma	499	492	498	499
Sarcoma	Sarcoma	265	257	263	247
Melanoma	Skin Cutaneous Melanoma	479	367	472	368
Stomach	Stomach Adenocarcinoma	478	441	415	395
Testicular germ cell	Testicular Germ Cell Cancer	156	150	156	155
Thymoma	Thymoma	124	123	120	123
Thyroid	Thyroid Carcinoma	516	499	509	405
Uterine CS	Uterine Carcinosarcoma	57	56	57	57
Uterine	Uterine Corpus Endometrial Carcinoma	548	539	177	248
Uveal melanoma	Uveal Melanoma	80	80	80	80
Total		11313	10845	9721	7978
