# Supplementary material for: Genotranscriptomic meta‐analysis of the CHD family chromatin remodelers in human cancers – initial evidence of an oncogenic role for CHD7
Source: Mol Oncol. 2017 Jul 21;11(10):1348–60. doi: 10.1002/1878-0261.12104 (PMC5623824; doi:10.1002/1878-0261.12104)
Supplement: Supplementary file 7 — Table S2. Frequency (%) of CHD genetic alterations in 32 tumor types from TCGA database. [file MOL2-11-1348-s007.pdf]

Table S2. Frequency (%) of CHD genetic alterations in 32 tumor types from TCGA database

Tumor Type	CHD1			CHD2			CHD3			CHD4			CHD5			CHD6			CHD7			CHD8			CHD9		
	A&G	Del	Mut	A&G	Del	Mut	A&G	Del	Mut	A&G	Del	Mut	A&G	Del	Mut	A&G	Del	Mut	A&G	Del	Mut	A&G	Del	Mut	A&G	Del	Mut
AML	0	7.3	0	0	2.1	0	0.5	7.3	0	0	1.6	1	2.6	1	0	0	2.1	0	12	0	0	1	0.5	0	0.5	2.1	0
ACC	67	3.3	0	14.8	23.3	3.3	6.8	38.9	1.1	72.2	4.4	1.1	5.6	42.2	2.2	57.8	3.3	0	41.1	13.3	3.3	31.1	20	1.1	55.6	6.7	1.1
Bladder	9.4	48.3	6.2	14.2	35	8.5	14.2	50.7	3.8	32.4	12.5	6.9	21.6	19.9	3.8	59.8	2.7	7.7	52.5	9.1	10	19.9	28.7	3.8	19.1	29.2	6.9
Glioma	1.1	7.8	0.3	2.8	10.3	0.3	3.9	3.1	0	13.5	3.9	0.7	2.1	41.3	0.7	8.8	0.6	0	9.4	2.5	0.3	2.3	13.3	0.7	3.5	4.1	0.7
Breast	18.5	25.8	0.6	17.3	24.5	1	5.6	60.4	1.4	25.2	14.2	2	7.3	40	1.4	42.2	6.7	1.9	51.9	6	1.4	18	22.5	1.4	14.2	55.7	1.4
Cervical	6.3	24.7	2.1	24.1	15.3	0.5	5.2	36.3	4.6	20	15.3	5.2	27.5	12.9	2.1	41.4	4.4	4.1	30.8	7.5	6.2	15.9	9.5	4.1	11.9	19.7	2.6
Cholangiocarcinoma	28.6	11.1	5.7	20	13.9	2.9	8.6	36.1	0	30.6	5.6	2.9	0	83.3	5.7	36.1	5.6	0	27.8	13.9	8.6	2.8	38.9	0	13.9	19.4	0
Colorectal	6.4	28.4	4	5.9	34.6	4.5	2.7	59.7	4.5	22.6	11.4	7.6	2.6	35.7	4.9	76.5	0.5	7.2	53.7	3.9	4	7.5	33.9	2.7	24.7	6.3	4.9
Esophagus	4.3	55.4	3.8	29.9	24.5	4.9	12	46.7	5.4	33.2	18.5	2.2	19	36.4	4.3	65.8	2.7	2.7	56	9.8	4.9	28.3	28.3	4.9	24.5	28.3	5.4
GBM	5.5	8	0.3	7	18.7	0.7	7	14.9	0.3	12.3	8.8	0.3	12.5	22.9	2.4	38.6	2.9	1	9.4	8.5	0.7	4.5	29.8	3.4	6.4	16.3	3.4
Head & neck	5.2	41.8	1.6	18.1	18.8	2.3	12.3	24.1	2.7	31.4	7.3	2.9	12.1	18	1.8	37.9	6.5	3.1	60.3	5	3.9	28.9	14.2	3.5	21.5	18.2	3.3
chRCC	12.1	15.2	0	33.3	3	1.5	0	75.8	3	31.8	1.5	3	1.5	80.3	0	31.8	4.5	0	27.3	15.2	1.5	31.8	3	0	31.8	7.6	0
ccRCC	44.4	1.7	1.3	5.4	6.8	1.6	4.9	9.7	2.9	23.3	0.9	2.2	1.7	19.3	1.1	22.3	0	1.1	12.1	14	2	3.4	39	0.9	18.6	4.2	0.7
pRCC	11.4	3.8	1.4	1.8	10.8	0.7	58.6	5.9	0.4	39.2	0.7	0.7	0.3	19.1	0.4	35.1	0.7	1.1	7.6	3.5	0.4	1	17.4	1.8	52.4	2.8	0.7
Liver	29.5	13	2.1	13.4	20.5	2.1	3.8	60.8	2.4	11.4	20.8	1.6	5.9	44.6	2.4	31.6	3	2.7	48.6	10	2.1	10.8	21.4	0.8	5.9	39.5	1.9
Lung adeno	18.7	40.3	0.9	13.5	40.7	1.3	6.5	53.5	2.2	28.3	25	2.6	23.8	22.9	4.3	39.5	15.9	5.7	48.8	10.1	5.2	32.9	19.6	5.2	20.7	27.5	4.3
Lung squ	2.3	77.8	3.4	35	19.6	2.3	6.8	62.5	1.7	50.7	7.6	3.4	12.6	43.3	4.5	52.7	9	4	51.9	12.8	8.5	28.3	34.7	4.5	18.8	37.1	4
DLBC	14.6	2.1	2.1	8.3	14.6	4.2	4.2	25	6.3	10.4	10.4	4.2	2.1	22.9	4.2	10.4	2.1	4.2	10.4	18.8	2.1	4.2	4.2	6.3	14.6	12.5	2.1
Mesothelioma	11.5	17.2	N/A	19.5	6.9	N/A	3.4	32.2	N/A	18.4	9.2	N/A	2.3	37.9	N/A	12.6	4.6	N/A	16.1	9.2	N/A	2.3	42.5	N/A	16.1	19.5	N/A
Ovarian	6.8	61.5	0.3	27.3	39.4	0.3	9	75	0.3	54.9	12.8	2.5	32.1	35.2	0.9	52.8	10.9	1.6	51.1	11.4	0.3	24.4	28.5	1.6	8.3	65.6	0.6
Pancreas	4	16.8	0	12.1	15.2	0.7	1.3	48.4	2	18.5	14.1	2	7.1	29.3	1.3	26.1	2.2	2.7	26.1	8.7	0.7	16.3	10.3	2	12.5	6.5	1.3
PCPG	4.3	7.4	0	8	2.5	1.1	0	39.5	1.1	7.4	1.2	0	0.6	63.6	0.5	6.2	1.9	0	8	7.4	0	2.5	13	0	4.3	3.7	0
Prostate	2	22.6	1.6	2.4	7.9	1.4	0.4	32.5	1.2	3	14.8	1.6	1.2	10.4	1.2	6.7	2.6	1.4	25	5.9	1.4	1.6	7.5	0.4	2.4	22.8	0.4
Sarcoma	28	16	0.8	32.9	15.6	1.6	21	41.2	0.8	17.5	30.7	0.4	28.4	24.1	2	38.9	5.8	1.6	31.1	12.5	2	29.2	21.8	1.2	5.8	55.3	0.8
Melanoma	12.5	27.2	3.8	33.1	10.4	5.2	10.8	36.8	4.1	18.5	15.8	7.3	20.4	21.8	9.2	49.9	1.9	11.1	46.6	7.9	8.7	11.7	30.2	6.8	11.2	32.2	6.5
Stomach	5.3	36.5	3.3	23.2	16.3	4.1	3.8	41	7.3	23.4	15.9	9.4	12.9	23.1	6.1	64.6	0.5	7.8	55.1	9.1	8.6	12	25.6	5.8	11.1	27	6.8
Testicular germ cell	3.4	69.3	0.6	32.2	21.3	0.6	25.5	29.3	0.6	98	0	2.6	16.7	22.7	1.3	28.7	8.7	1.3	81.3	2.7	0	34	13.3	0.6	12	34	0.6
Thymoma	7.3	1.6	0	10.6	1.6	0.8	1.6	12.2	1.6	3.3	6.5	0	0.8	6.5	0	8.1	0.8	0	8.1	1.6	0	10.6	1.6	0.8	1.6	10.6	0.8
Thyroid	3.3	0	0	0	1.6	0.5	3.5	1.6	0.7	2.6	0.2	0.2	0.4	1	0	2.4	0	0.2	1.4	0.8	0.2	2.2	0.2	0	2.6	0.4	0.7
Uterine CS	10.7	46.4	3.5	17.9	50	1.8	8.9	75	0	39.3	23.2	17.5	37.5	30.4	5.3	80.4	5.4	1.8	64.3	12.5	3.5	25	46.4	1.8	12.5	67.9	1.8
Uterine	3.7	18.7	4.4	5.4	17.3	6	2.1	25.6	10.5	17.3	7.2	14.1	9.5	13	8.5	25.6	2.4	8.1	31.9	2.6	8.1	10.4	11.3	7.7	3.7	25.4	6.9
Uveal melanoma	3.8	3.8	0	2.5	5	0	10	2.5	0	3.8	3.8	0	0	30	0	11.3	0	0	62.5	0	0	3.8	1.3	0	1.3	20	0

Note: A & G: Amplification and Gain; Del; Deletion; Mut: Mutation
