# Supplementary material for: Genotranscriptomic meta‐analysis of the CHD family chromatin remodelers in human cancers – initial evidence of an oncogenic role for CHD7
Source: Mol Oncol. 2017 Jul 21;11(10):1348–60. doi: 10.1002/1878-0261.12104 (PMC5623824; doi:10.1002/1878-0261.12104)
Supplement: Supplementary file 8 — Table S3. Mutations of CHD6 in TCGA tumors. [file MOL2-11-1348-s008.pdf]

Table S3. Mutations of CHD6 in TCGA Tumors

Sample ID	Cancer Type	AA change	Type	Mutation Assessor
TCGA-BT-A3PK-01	Bladder	G510V	Missense	High
TCGA-A8-A0A6-01	Breast	V701G	Missense	High
TCGA-KU-A66S-01	Head & neck	P629R	Missense	High
TCGA-CV-7245-01	Head & neck	D886N	Missense	High
TCGA-DD-A39Y-01	Liver	E951G	Missense	High
TCGA-67-3771-01	Lung adeno	P517S	Missense	High
TCGA-IB-7651-01	Pancreas	E487G	Missense	High
TCGA-2J-AAB1-01	Pancreas	E914K	Missense	High
TCGA-EE-A2MJ-06	Melanoma	P517S	Missense	High
TCGA-BR-8591-01	Stomach	R681L	Missense	High
TCGA-HU-A4H4-01	Stomach	T493A	Missense	High
TCGA-AP-A051-01	Uterine	A891T	Missense	High
TCGA-FD-A3SN-01	Bladder	P1896T	Missense	Low
TCGA-GC-A3I6-01	Bladder	Q1647E	Missense	Low
TCGA-DK-A1AG-01	Bladder	E1998Q	Missense	Low
TCGA-GV-A3JW-01	Bladder	D1910H	Missense	Low
TCGA-FJ-A3ZE-01	Bladder	K2572N	Missense	Low
TCGA-K4-A3WS-01	Bladder	R2035T	Missense	Low
TCGA-GC-A3RC-01	Bladder	S2220C	Missense	Low
TCGA-BH-A1EY-01	Breast	S230C	Missense	Low
TCGA-GI-A2C8-01	Breast	E487D	Missense	Low
TCGA-D8-A1J8-01	Breast	E2676K	Missense	Low
TCGA-E9-A5FL-01	Breast	E2016K	Missense	Low
TCGA-BH-A0BR-01	Breast	K4T	Missense	Low
TCGA-EW-A1P8-01	Breast	P1924S	Missense	Low
TCGA-A7-A0CJ-01	Breast	K134N	Missense	Low
TCGA-AN-A046-01	Breast	D813N	Missense	Low
TCGA-EA-A3HU-01	Cervical	D813N	Missense	Low
TCGA-JX-A3Q0-01	Cervical	L2420F	Missense	Low
TCGA-DR-A0ZM-01	Cervical	E2585K	Missense	Low
TCGA-EK-A3GK-01	Cervical	R2406T	Missense	Low
TCGA-EK-A2RA-01	Cervical	G2213R	Missense	Low
TCGA-AA-3696-01	Colorectal	D661N	Missense	Low
TCGA-AA-3845-01	Colorectal	E66D	Missense	Low
TCGA-AA-3672-01	Colorectal	V572I	Missense	Low
TCGA-AA-A010-01	Colorectal	F1823C	Missense	Low
TCGA-AA-3864-01	Colorectal	A345T	Missense	Low
TCGA-AA-3956-01	Colorectal	E1765Q	Missense	Low
TCGA-AA-3811-01	Colorectal	E1751D	Missense	Low
TCGA-AA-3710-01	Colorectal	E2120V	Missense	Low
TCGA-AA-3833-01	Colorectal	L216P	Missense	Low
TCGA-JY-A6FG-01	Esophagus	A2543E	Missense	Low
TCGA-76-4928-01	GBM	Q1353P	Missense	Low
TCGA-P3-A6T7-01	Head & neck	L266V	Missense	Low
TCGA-CV-7252-01	Head & neck	H1863Y	Missense	Low
TCGA-DD-AAEG-01	Liver	E45Q	Missense	Low
TCGA-DD-A113-01	Liver	D1841V	Missense	Low
TCGA-CC-A7IL-01	Liver	H2040Y	Missense	Low
TCGA-DD-A3A8-01	Liver	H1993L	Missense	Low
TCGA-50-5933-01	Lung adeno	Q2078H	Missense	Low
TCGA-73-7499-01	Lung adeno	E1918Q	Missense	Low
TCGA-44-2656-01	Lung adeno	Y1092F	Missense	Low
TCGA-78-7155-01	Lung adeno	R1928M	Missense	Low
TCGA-22-4604-01	Lung squ	N1344I	Missense	Low
TCGA-66-2787-01	Lung squ	R1296K	Missense	Low
TCGA-G8-6326-01	DLBC	A1205G	Missense	Low
TCGA-G8-6326-01	DLBC	H1942L	Missense	Low
TCGA-25-1317-01	Ovarian	K949E	Missense	Low
TCGA-IB-7651-01	Pancreas	A2552V	Missense	Low

TCGA-XK-AAIW-01	Prostate	C2685Y	Missense	Low
TCGA-DX-AB2E-01	Sarcoma	T2568I	Missense	Low
TCGA-QC-A7B5-01	Sarcoma	S1719F	Missense	Low
TCGA-DX-A6BA-01	Sarcoma	P2590R	Missense	Low
TCGA-QC-A7B5-01	Sarcoma	I1484M	Missense	Low
TCGA-DX-AB2E-01	Sarcoma	G81E	Missense	Low
TCGA-EE-A20C-06	Melanoma	P2143S	Missense	Low
TCGA-EE-A29R-06	Melanoma	P312L	Missense	Low
TCGA-EB-A4IS-01	Melanoma	P2354S	Missense	Low
TCGA-FS-A1ZF-06	Melanoma	E123K	Missense	Low
TCGA-FW-A5DX-01	Melanoma	P1383Q	Missense	Low
TCGA-FS-A4F5-06	Melanoma	D2663N	Missense	Low
TCGA-EB-A5UL-06	Melanoma	S277F	Missense	Low
TCGA-FS-A1ZK-06	Melanoma	S1359F	Missense	Low
TCGA-FR-A726-01	Melanoma	L2420F	Missense	Low
TCGA-EE-A2M5-06	Melanoma	E46K	Missense	Low
TCGA-D9-A6EC-06	Melanoma	D258V	Missense	Low
TCGA-EE-A181-06	Melanoma	S1059L	Missense	Low
TCGA-EE-A3AG-06	Melanoma	P2329S	Missense	Low
TCGA-ER-A1A1-06	Melanoma	A268T	Missense	Low
TCGA-EE-A29D-06	Melanoma	P1521S	Missense	Low
TCGA-EB-A44O-01	Melanoma	P2421S	Missense	Low
TCGA-FS-A1ZB-06	Melanoma	P2439L	Missense	Low
TCGA-FW-A3R5-06	Melanoma	P2491L	Missense	Low
TCGA-D9-A6EC-06	Melanoma	M739L	Missense	Low
TCGA-CG-5721-01	Stomach	A2251T	Missense	Low
TCGA-BR-4201-01	Stomach	K1055T	Missense	Low
TCGA-BR-8487-01	Stomach	P732S	Missense	Low
TCGA-BR-A4QL-01	Stomach	A2158V	Missense	Low
TCGA-BR-6452-01	Stomach	R915C	Missense	Low
TCGA-CD-8535-01	Stomach	N1973K	Missense	Low
TCGA-FP-8631-01	Stomach	E2163D	Missense	Low
TCGA-BR-A4J4-01	Stomach	D2571N	Missense	Low
TCGA-BR-7716-01	Stomach	K424T	Missense	Low
TCGA-VQ-A8P2-01	Stomach	R810H	Missense	Low
TCGA-HF-A5NB-01	Stomach	M2254I	Missense	Low
TCGA-HU-A4GX-01	Stomach	A2310V	Missense	Low
TCGA-BR-4362-01	Stomach	E2005Q	Missense	Low
TCGA-BR-6454-01	Stomach	E1951K	Missense	Low
TCGA-2G-AAKL-01	Testicular germ cell	N2715S	Missense	Low
TCGA-AX-A0J0-01	Uterine	E46D	Missense	Low
TCGA-BS-A0UF-01	Uterine	I1690S	Missense	Low
TCGA-B5-A11Y-01	Uterine	M2505I	Missense	Low
TCGA-BS-A0UF-01	Uterine	E1704D	Missense	Low
TCGA-BS-A0UJ-01	Uterine	I2622V	Missense	Low
TCGA-DI-A0WH-01	Uterine	T2504M	Missense	Low
TCGA-AP-A0LM-01	Uterine	L1125M	Missense	Low
TCGA-D1-A160-01	Uterine	E1417D	Missense	Low
TCGA-AP-A051-01	Uterine	P2551L	Missense	Low
TCGA-AP-A051-01	Uterine	P1700H	Missense	Low
TCGA-K4-A3WS-01	Bladder	V1462M	Missense	Medium
TCGA-BT-A3PK-01	Bladder	G2620V	Missense	Medium
TCGA-A7-A0CD-01	Breast	P1604R	Missense	Medium
TCGA-LL-A5YP-01	Breast	M2520I	Missense	Medium
TCGA-A2-A0T5-01	Breast	W1112G	Missense	Medium
TCGA-GM-A2DO-01	Breast	I294M	Missense	Medium
TCGA-EK-A3GM-01	Cervical	R2072Q	Missense	Medium
TCGA-EK-A2RJ-01	Cervical	E2295K	Missense	Medium
TCGA-EA-A5FO-01	Cervical	R240K	Missense	Medium
TCGA-AG-A002-01	Colorectal	D417G	Missense	Medium
TCGA-AA-3710-01	Colorectal	R1301Q	Missense	Medium
TCGA-AA-3672-01	Colorectal	Y319C	Missense	Medium

TCGA-AA-3984-01	Colorectal	F1154L	Missense	Medium
TCGA-AG-A015-01	Colorectal	R1434S	Missense	Medium
TCGA-JY-A93D-01	Esophagus	G2062R	Missense	Medium
TCGA-L5-A8NM-01	Esophagus	R1482C	Missense	Medium
TCGA-06-0241-01	GBM	P2126S	Missense	Medium
TCGA-UF-A7JT-01	Head & neck	E450K	Missense	Medium
TCGA-CV-A6JY-01	Head & neck	L2101V	Missense	Medium
TCGA-CV-A6JM-01	Head & neck	S2115F	Missense	Medium
TCGA-CN-6023-01	Head & neck	P2393R	Missense	Medium
TCGA-CJ-4899-01	ccRCC	G2494E	Missense	Medium
TCGA-B0-4815-01	ccRCC	E337G	Missense	Medium
TCGA-B0-5098-01	ccRCC	D1063G	Missense	Medium
TCGA-AK-3428-01	ccRCC	D1074H	Missense	Medium
TCGA-A4-7286-01	pRCC	W1156S	Missense	Medium
TCGA-BQ-7056-01	pRCC	C1408W	Missense	Medium
TCGA-DD-A3A1-01	Liver	R1453G	Missense	Medium
TCGA-95-7567-01	Lung adeno	P1273L	Missense	Medium
TCGA-44-4112-01	Lung adeno	D366G	Missense	Medium
TCGA-49-4486-01	Lung adeno	D417N	Missense	Medium
TCGA-44-6776-01	Lung adeno	W1575L	Missense	Medium
TCGA-39-5036-01	Lung squ	R1135H	Missense	Medium
TCGA-46-6025-01	Lung squ	R2287L	Missense	Medium
TCGA-39-5031-01	Lung squ	P2045S	Missense	Medium
TCGA-13-0755-01	Ovarian	V1289M	Missense	Medium
TCGA-24-1105-01	Ovarian	Y1570C	Missense	Medium
TCGA-61-1995-01	Ovarian	R440Q	Missense	Medium
TCGA-IB-7651-01	Pancreas	A1504D	Missense	Medium
TCGA-2J-AABR-01	Pancreas	R1482C	Missense	Medium
TCGA-2L-AAQE-01	Pancreas	R1482C	Missense	Medium
TCGA-ZG-A8QY-01	Prostate	I344M	Missense	Medium
TCGA-TK-A8OK-01	Prostate	I294T	Missense	Medium
TCGA-XK-AAIW-01	Prostate	Y1237H	Missense	Medium
TCGA-EE-A2GC-06	Melanoma	S720F	Missense	Medium
TCGA-QB-A6FS-06	Melanoma	S720F	Missense	Medium
TCGA-EE-A3J4-06	Melanoma	L2613R	Missense	Medium
TCGA-BF-A5EQ-01	Melanoma	P2466L	Missense	Medium
TCGA-DA-A1I1-06	Melanoma	R1135H	Missense	Medium
TCGA-EE-A3AE-06	Melanoma	S2594F	Missense	Medium
TCGA-FR-A3YO-06	Melanoma	P646S	Missense	Medium
TCGA-EE-A2GI-06	Melanoma	P1079L	Missense	Medium
TCGA-EE-A2GS-06	Melanoma	P1181S	Missense	Medium
TCGA-FR-A726-01	Melanoma	S999F	Missense	Medium
TCGA-D3-A2J8-06	Melanoma	D1492N	Missense	Medium
TCGA-IH-A3EA-01	Melanoma	E427G	Missense	Medium
TCGA-FS-A1ZZ-06	Melanoma	P2499S	Missense	Medium
TCGA-EE-A3AA-06	Melanoma	R343C	Missense	Medium
TCGA-EE-A2A2-06	Melanoma	G1186E	Missense	Medium
TCGA-D9-A4Z3-01	Melanoma	S1485F	Missense	Medium
TCGA-D3-A5GO-06	Melanoma	S1485F	Missense	Medium
TCGA-CG-4300-01	Stomach	L2109V	Missense	Medium
TCGA-HU-A4G8-01	Stomach	Q1624H	Missense	Medium
TCGA-BR-4184-01	Stomach	L1553F	Missense	Medium
TCGA-BR-4184-01	Stomach	R810C	Missense	Medium
TCGA-CG-5726-01	Stomach	G392V	Missense	Medium
TCGA-HU-A4GT-01	Stomach	Y1140C	Missense	Medium
TCGA-BR-7707-01	Stomach	Y1057C	Missense	Medium
TCGA-CG-4305-01	Stomach	R834W	Missense	Medium
TCGA-HU-A4GU-01	Stomach	E1335K	Missense	Medium
TCGA-CG-4437-01	Stomach	E2214K	Missense	Medium
TCGA-IN-A6RN-01	Stomach	Y1633H	Missense	Medium
TCGA-BJ-A4O9-01	Thyroid	N2470S	Missense	Medium
TCGA-D1-A160-01	Uterine	G2494W	Missense	Medium

TCGA-D1-A103-01	Uterine	N324H	Missense	Medium
TCGA-AX-A0J1-01	Uterine	R1541H	Missense	Medium
TCGA-B5-A0JY-01	Uterine	F325V	Missense	Medium
TCGA-AP-A059-01	Uterine	V1312D	Missense	Medium
TCGA-AX-A0J1-01	Uterine	V786A	Missense	Medium
TCGA-D1-A16J-01	Uterine	E1280K	Missense	Medium
TCGA-BG-A0MO-01	Uterine	W1388C	Missense	Medium
TCGA-AP-A051-01	Uterine	L1222I	Missense	Medium
TCGA-B5-A11I-01	Uterine	R1397G	Missense	Medium
TCGA-B5-A0JY-01	Uterine	K1473N	Missense	Medium
TCGA-D1-A17Q-01	Uterine	L791R	Missense	Medium
TCGA-D1-A15X-01	Uterine	G1587S	Missense	Medium
TCGA-B5-A0JY-01	Uterine	C1631R	Missense	Medium
TCGA-GV-A3JZ-01	Bladder	E305K	Missense	Neutral
TCGA-AC-A23H-01	Breast	K387E	Missense	Neutral
TCGA-AA-3811-01	Colorectal	R222W	Missense	Neutral
TCGA-AG-A01W-01	Colorectal	G798D	Missense	Neutral
TCGA-AA-A01Q-01	Colorectal	A1976T	Missense	Neutral
TCGA-L5-A4OH-01	Esophagus	N562K	Missense	Neutral
TCGA-IG-A4P3-01	Esophagus	I2160V	Missense	Neutral
TCGA-14-1456-01	GBM	C1832Y	Missense	Neutral
TCGA-CN-6995-01	Head & neck	S2667F	Missense	Neutral
TCGA-P3-A6T5-01	Head & neck	E141K	Missense	Neutral
TCGA-CV-7242-01	Head & neck	K480R	Missense	Neutral
TCGA-RC-A6M3-01	Liver	R915H	Missense	Neutral
TCGA-BC-A5W4-01	Liver	A2158T	Missense	Neutral
TCGA-CC-A8HT-01	Liver	T1588A	Missense	Neutral
TCGA-49-4487-01	Lung adeno	G88R	Missense	Neutral
TCGA-55-7574-01	Lung adeno	K1370N	Missense	Neutral
TCGA-18-4086-01	Lung squ	A1932S	Missense	Neutral
TCGA-G8-6909-01	DLBC	N1899I	Missense	Neutral
TCGA-24-1553-01	Ovarian	R1091H	Missense	Neutral
TCGA-IB-7651-01	Pancreas	S1738L	Missense	Neutral
TCGA-IB-7651-01	Pancreas	A2157V	Missense	Neutral
TCGA-CH-5765-01	Prostate	N29S	Missense	Neutral
TCGA-EE-A2MR-06	Melanoma	P2540L	Missense	Neutral
TCGA-EE-A2MU-06	Melanoma	K1411R	Missense	Neutral
TCGA-EE-A2GD-06	Melanoma	A891D	Missense	Neutral
TCGA-FW-A3R5-06	Melanoma	T737I	Missense	Neutral
TCGA-BR-8361-01	Stomach	F499L	Missense	Neutral
TCGA-BR-6452-01	Stomach	N1905S	Missense	Neutral
TCGA-D7-6528-01	Stomach	V2564A	Missense	Neutral
TCGA-CG-5726-01	Stomach	R222W	Missense	Neutral
TCGA-2G-AALO-01	Testicular germ cell	Q447K	Missense	Neutral
TCGA-AP-A056-01	Uterine	A69T	Missense	Neutral
TCGA-BS-A0UF-01	Uterine	R832Q	Missense	Neutral
TCGA-AP-A059-01	Uterine	A2569V	Missense	Neutral
TCGA-AP-A051-01	Uterine	D1742Y	Missense	Neutral
TCGA-D1-A103-01	Uterine	S2667P	Missense	Neutral
TCGA-D1-A103-01	Uterine	A2158T	Missense	Neutral
TCGA-B5-A0JY-01	Uterine	A1811T	Missense	Neutral
TCGA-DK-A1A3-01	Bladder	X1336_splice	Splice	
TCGA-E2-A10C-01	Breast	R2137*	Nonsense	
TCGA-D8-A1JP-01	Breast	Y1237*	Nonsense	
TCGA-BH-A0H7-01	Breast	R350*	Nonsense	
TCGA-AR-A24O-01	Breast	X1336_splice	Splice	
TCGA-E2-A109-01	Breast	L298Rfs*61	FS del	
TCGA-AA-3715-01	Colorectal	R236*	Nonsense	
TCGA-AA-3966-01	Colorectal	R763*	Nonsense	
TCGA-CV-A461-01	Head & neck	G1245*	Nonsense	
TCGA-F7-A624-01	Head & neck	X1336_splice	Splice	
TCGA-CV-5442-01	Head & neck	T1356T	splice_region	

TCGA-QK-A6IJ-01	Head & neck	X2377_splice	Splice
TCGA-CR-5248-01	Head & neck	R2137*	Nonsense
TCGA-BP-4994-01	ccRCC	K1152Efs*63	FS del
TCGA-UZ-A9PL-01	pRCC	Y905*	Nonsense
TCGA-G3-A3CJ-01	Liver	E2704Rfs*18	FS del
TCGA-G3-A3CJ-01	Liver	F1001Lfs*17	FS del
TCGA-49-6761-01	Lung adeno	G87*	Nonsense
TCGA-95-7043-01	Lung adeno	G627*	Nonsense
TCGA-66-2786-01	Lung squ	R2137*	Nonsense
TCGA-IB-7651-01	Pancreas	R1301*	Nonsense
TCGA-IB-7651-01	Pancreas	X823_splice	Splice
TCGA-KK-A59Y-01	Prostate	K243_R244insLK	IF ins
TCGA-XQ-A8TB-01	Prostate	E1874_N1877del	IF del
TCGA-XK-AAIW-01	Prostate	R832*	Nonsense
TCGA-CH-5767-01	Prostate	N481Kfs*6	FS ins
TCGA-IE-A6BZ-01	Sarcoma	X2163_splice	Splice
TCGA-EE-A29N-06	Melanoma	W446*	Nonsense
TCGA-EE-A29E-06	Melanoma	W1112*	Nonsense
TCGA-EB-A431-01	Melanoma	Q1687*	Nonsense
TCGA-EE-A29N-06	Melanoma	W446*	Nonsense
TCGA-BR-7703-01	Stomach	X753_splice	Splice
TCGA-R5-A7ZF-01	Stomach	[Not Available]	splice_region
TCGA-BR-4257-01	Stomach	[Not Available]	splice_region
TCGA-N5-A4RV-01	Uterine CS	E696Dfs*4	FS del
TCGA-AX-A05Z-01	Uterine	R350*	Nonsense
TCGA-AX-A05Z-01	Uterine	E1998*	Nonsense
TCGA-AP-A0LM-01	Uterine	R1232*	Nonsense
TCGA-A5-A0GJ-01	Uterine	R2447Pfs*15	FS del
TCGA-AX-A0J0-01	Uterine	S1738*	Nonsense
