# Supplementary material for: Genotranscriptomic meta‐analysis of the CHD family chromatin remodelers in human cancers – initial evidence of an oncogenic role for CHD7
Source: Mol Oncol. 2017 Jul 21;11(10):1348–60. doi: 10.1002/1878-0261.12104 (PMC5623824; doi:10.1002/1878-0261.12104)
Supplement: Supplementary file 9 — Table S4. Mutations of CHD7 in TCGATumors. [file MOL2-11-1348-s009.pdf]

Table S4. Mutations of CHD7 in TCGATumors

Sample ID	Cancer Type	AA change	Type	Mutation Assessor
TCGA-39-5037-01	Lung squ	G1048W	Missense	High
TCGA-AA-3672-01	Colorectal	T917M3D	Missense	High
TCGA-AA-A01K-01	Colorectal	R1189H	Missense	High
TCGA-BS-A0WQ-01	Uterine	T1133M	Missense	High
TCGA-IB-7651-01	Pancreas	A1428D3D	Missense	High
TCGA-LP-A5U2-01	Cervical	R1402T3D	Missense	High
TCGA-T2-A6X2-01	Head & neck	E1106K	Missense	High
TCGA-06-0211-02	GBM	S2535N	Missense	Low
TCGA-06-0877-01	GBM	Q1928L	Missense	Low
TCGA-18-3419-01	Lung squ	E2172K	Missense	Low
TCGA-22-4601-01	Lung squ	K713T	Missense	Low
TCGA-22-5477-01	Lung squ	S355P	Missense	Low
TCGA-33-4532-01	Lung squ	P405L	Missense	Low
TCGA-33-4532-01	Lung squ	E2412Q	Missense	Low
TCGA-43-6143-01	Lung squ	F2268L	Missense	Low
TCGA-66-2787-01	Lung squ	K654N	Missense	Low
TCGA-66-2788-01	Lung squ	S103L	Missense	Low
TCGA-70-6722-01	Lung squ	N2817I	Missense	Low
TCGA-75-6214-01	Lung adeno	K1301E3D	Missense	Low
TCGA-78-7163-01	Lung adeno	D2249N	Missense	Low
TCGA-78-7536-01	Lung adeno	S1604C	Missense	Low
TCGA-95-7947-01	Lung adeno	D952N	Missense	Low
TCGA-A7-A26E-01	Breast	G128V	Missense	Low
TCGA-A7-A26H-01	Breast	E2191Q	Missense	Low
TCGA-A7-A26I-01	Breast	P2586S3D	Missense	Low
TCGA-A8-A09Z-01	Breast	Y75H	Missense	Low
TCGA-A8-A0A6-01	Breast	H368P	Missense	Low
TCGA-AA-3949-01	Colorectal	I1500V	Missense	Low
TCGA-AG-A002-01	Colorectal	K597N	Missense	Low
TCGA-AP-A051-01	Uterine	R2400W	Missense	Low
TCGA-AP-A051-01	Uterine	A2876D	Missense	Low
TCGA-AP-A056-01	Uterine	F2458C	Missense	Low
TCGA-AP-A056-01	Uterine	R312Q	Missense	Low
TCGA-AP-A05N-01	Uterine	E1423G3D	Missense	Low
TCGA-AP-A0LE-01	Uterine	Q2338P	Missense	Low
TCGA-AP-A0LM-01	Uterine	P1705L	Missense	Low
TCGA-AP-A0LT-01	Uterine	A2930V	Missense	Low
TCGA-B0-5109-01	ccRCC	N2817S	Missense	Low
TCGA-B0-5117-01	ccRCC	D2291E	Missense	Low
TCGA-B5-A0JY-01	Uterine	L2574I3D	Missense	Low
TCGA-B5-A11E-01	Uterine	R2398H	Missense	Low
TCGA-B5-A11E-01	Uterine	S268F	Missense	Low
TCGA-B5-A11E-01	Uterine	P2558S	Missense	Low
TCGA-BF-A1PZ-01	Melanoma	S2142F	Missense	Low
TCGA-BF-A3DJ-01	Melanoma	K713R	Missense	Low
TCGA-BH-A0DZ-01	Breast	Y72C	Missense	Low
TCGA-BH-A0HF-01	Breast	D2443A	Missense	Low
TCGA-BR-4253-01	Stomach	P366S	Missense	Low
TCGA-BR-4362-01	Stomach	R1939Q	Missense	Low
TCGA-BR-4368-01	Stomach	S466L	Missense	Low
TCGA-BR-4371-01	Stomach	D2957N	Missense	Low
TCGA-BR-8059-01	Stomach	P30L	Missense	Low
TCGA-BR-8592-01	Stomach	R250C	Missense	Low
TCGA-BS-A0UJ-01	Uterine	D1878N	Missense	Low
TCGA-BT-A3PH-01	Bladder	E12K	Missense	Low
TCGA-CD-5800-01	Stomach	E2222G	Missense	Low
TCGA-CD-8534-01	Stomach	L563V	Missense	Low
TCGA-CG-4305-01	Stomach	R1939Q	Missense	Low
TCGA-CG-4305-01	Stomach	P260L	Missense	Low
TCGA-CG-5721-01	Stomach	E1209D	Missense	Low
TCGA-CJ-5681-01	ccRCC	R1608K	Missense	Low

TCGA-CQ-A4C9-01	Head & neck	E2959K	Missense	Low
TCGA-CR-7364-01	Head & neck	H2628R	Missense	Low
TCGA-CV-5439-01	Head & neck	A391V	Missense	Low
TCGA-CV-6954-01	Head & neck	T2738M	Missense	Low
TCGA-D1-A15X-01	Uterine	N1237D	Missense	Low
TCGA-D3-A1QA-06	Melanoma	S2501F	Missense	Low
TCGA-D3-A2JO-06	Melanoma	S236F	Missense	Low
TCGA-DA-A1HV-06	Melanoma	P515S	Missense	Low
TCGA-DD-A4NE-01	Liver	R1351K3D	Missense	Low
TCGA-DR-A0ZM-01	Cervical	D2033H	Missense	Low
TCGA-DU-7306-01	Glioma	E22Q	Missense	Low
TCGA-EA-A410-01	Cervical	R2402G	Missense	Low
TCGA-EB-A41A-01	Melanoma	G97E	Missense	Low
TCGA-EB-A41A-01	Melanoma	G97R	Missense	Low
TCGA-EB-A41A-01	Melanoma	P2257L	Missense	Low
TCGA-EE-A29D-06	Melanoma	S2906F	Missense	Low
TCGA-EE-A2A2-06	Melanoma	S268F	Missense	Low
TCGA-EE-A2GN-06	Melanoma	R1445T3D	Missense	Low
TCGA-EE-A2MJ-06	Melanoma	S2237F	Missense	Low
TCGA-EE-A2MR-06	Melanoma	S2158L	Missense	Low
TCGA-EE-A3AG-06	Melanoma	P354S	Missense	Low
TCGA-EK-A3GN-01	Cervical	P568L	Missense	Low
TCGA-EP-A2KA-01	Liver	A2104V	Missense	Low
TCGA-ER-A19P-06	Melanoma	G1845R	Missense	Low
TCGA-F1-A448-01	Stomach	G451S	Missense	Low
TCGA-F7-8489-01	Head & neck	Y759F	Missense	Low
TCGA-F7-A624-01	Head & neck	A2726T	Missense	Low
TCGA-FC-7708-01	Prostate	M2799I	Missense	Low
TCGA-FP-A4BE-01	Stomach	T2738M	Missense	Low
TCGA-FP-A4BE-01	Stomach	P86L	Missense	Low
TCGA-FR-A726-01	Melanoma	P621L	Missense	Low
TCGA-FV-A2QR-01	Liver	P732S	Missense	Low
TCGA-FW-A3R5-06	Melanoma	P2819S	Missense	Low
TCGA-FW-A3R5-06	Melanoma	S2490L	Missense	Low
TCGA-FW-A5DX-01	Melanoma	S2308L	Missense	Low
TCGA-G2-A3VY-01	Bladder	P109T	Missense	Low
TCGA-GC-A3BM-01	Bladder	T760A	Missense	Low
TCGA-GD-A3OP-01	Bladder	G1014E	Missense	Low
TCGA-GN-A266-06	Melanoma	L2498F	Missense	Low
TCGA-H4-A2HQ-01	Bladder	S465Y	Missense	Low
TCGA-H7-7774-01	Head & neck	R2098Q	Missense	Low
TCGA-HD-7832-01	Head & neck	S2142F	Missense	Low
TCGA-HF-7132-01	Stomach	E2408K	Missense	Low
TCGA-HF-A5NB-01	Stomach	Y2601C3D	Missense	Low
TCGA-HU-8243-01	Stomach	Q359E	Missense	Low
TCGA-HU-8244-01	Stomach	K683R	Missense	Low
TCGA-HU-A4GN-01	Stomach	P287L	Missense	Low
TCGA-IB-7651-01	Pancreas	I1311V3D	Missense	Low
TCGA-IG-A8O2-01	Esophagus	K1536R	Missense	Low
TCGA-IQ-A61H-01	Head & neck	P101L	Missense	Low
TCGA-KN-8428-01	chRCC	E2253G	Missense	Low
TCGA-KR-A7K2-01	Liver	H106Y	Missense	Low
TCGA-L5-A4OH-01	Esophagus	R1219L	Missense	Low
TCGA-L5-A88W-01	Esophagus	E1478K	Missense	Low
TCGA-L5-A8NF-01	Esophagus	F1799S	Missense	Low
TCGA-LP-A5U2-01	Cervical	E1478K	Missense	Low
TCGA-MY-A5BD-01	Cervical	P674L	Missense	Low
TCGA-N8-A56S-01	Uterine CS	R2653Q3D	Missense	Low
TCGA-OR-A5KB-01	ACC	D2126H	Missense	Low
TCGA-OR-A5KB-01	ACC	A149S	Missense	Low
TCGA-P6-A5OF-01	ACC	P2493R	Missense	Low
TCGA-Q1-A5R2-01	Cervical	G25E	Missense	Low
TCGA-R6-A6Y0-01	Esophagus	Q320E	Missense	Low
TCGA-VQ-A8P2-01	Stomach	G446R	Missense	Low

TCGA-VQ-A8P2-01	Stomach	P287S	Missense	Low
TCGA-W5-AA34-01	Cholangiocarcinoma	P153Q	Missense	Low
TCGA-X6-A7WD-01	Sarcoma	V424F	Missense	Low
TCGA-18-3415-01	Lung squ	L1007F	Missense	Medium
TCGA-21-5782-01	Lung squ	K1925E	Missense	Medium
TCGA-21-5782-01	Lung squ	R1593L	Missense	Medium
TCGA-49-6745-01	Lung adeno	D1578N	Missense	Medium
TCGA-66-2755-01	Lung squ	R2023M	Missense	Medium
TCGA-78-7158-01	Lung adeno	I1295N3D	Missense	Medium
TCGA-AA-3984-01	Colorectal	K2331R	Missense	Medium
TCGA-AA-A004-01	Colorectal	R1493H	Missense	Medium
TCGA-AA-A010-01	Colorectal	K1264T3D	Missense	Medium
TCGA-AK-3430-01	ccRCC	R1820Q	Missense	Medium
TCGA-AP-A059-01	Uterine	R1976C	Missense	Medium
TCGA-AP-A059-01	Uterine	L2749M	Missense	Medium
TCGA-AP-A0LM-01	Uterine	R1914H	Missense	Medium
TCGA-AX-A05Z-01	Uterine	R851I	Missense	Medium
TCGA-AX-A0J0-01	Uterine	D849Y	Missense	Medium
TCGA-B0-4813-01	ccRCC	S1874P	Missense	Medium
TCGA-B0-4823-01	ccRCC	L1794I	Missense	Medium
TCGA-B5-A0JY-01	Uterine	F826L	Missense	Medium
TCGA-B5-A11E-01	Uterine	A1535T	Missense	Medium
TCGA-BA-A6DA-01	Head & neck	D1296H3D	Missense	Medium
TCGA-BF-A5ES-01	Melanoma	R1976C	Missense	Medium
TCGA-BP-4807-01	ccRCC	L1229V	Missense	Medium
TCGA-BP-5006-01	ccRCC	C2324Y	Missense	Medium
TCGA-BR-4361-01	Stomach	H2071R	Missense	Medium
TCGA-BR-4362-01	Stomach	K1116E	Missense	Medium
TCGA-BR-6452-01	Stomach	R1914H	Missense	Medium
TCGA-BS-A0UF-01	Uterine	K1196N	Missense	Medium
TCGA-BS-A0UF-01	Uterine	K2013T	Missense	Medium
TCGA-BT-A3PJ-01	Bladder	E1580K	Missense	Medium
TCGA-C5-A1MK-01	Cervical	D1993H	Missense	Medium
TCGA-CC-A3MB-01	Liver	L1647V	Missense	Medium
TCGA-CG-4460-01	Stomach	R1632H	Missense	Medium
TCGA-CN-6988-01	Head & neck	L1909M	Missense	Medium
TCGA-CV-5970-01	Head & neck	E2009K	Missense	Medium
TCGA-CV-A461-01	Head & neck	D1623H	Missense	Medium
TCGA-D1-A103-01	Uterine	F826L	Missense	Medium
TCGA-D3-A5GO-06	Melanoma	S2123F	Missense	Medium
TCGA-DD-A39Z-01	Liver	E1658G	Missense	Medium
TCGA-DK-A1AD-01	Bladder	W1966C	Missense	Medium
TCGA-EA-A3HU-01	Cervical	L1464V3D	Missense	Medium
TCGA-EB-A4XL-01	Melanoma	S1907L	Missense	Medium
TCGA-EB-A6QY-01	Melanoma	D1090N	Missense	Medium
TCGA-EE-A29L-06	Melanoma	E1060K	Missense	Medium
TCGA-EE-A2MP-06	Melanoma	R886Q3D	Missense	Medium
TCGA-EE-A2MR-06	Melanoma	S1357F3D	Missense	Medium
TCGA-EE-A3JI-06	Melanoma	P1300S3D	Missense	Medium
TCGA-EJ-A65F-01	Prostate	P940L	Missense	Medium
TCGA-EP-A2KB-01	Liver	R1620L	Missense	Medium
TCGA-EP-A2KB-01	Liver	R2057S	Missense	Medium
TCGA-ER-A194-01	Melanoma	G1982E	Missense	Medium
TCGA-ER-A3PL-06	Melanoma	G1619R	Missense	Medium
TCGA-ER-A3PL-06	Melanoma	G1619E	Missense	Medium
TCGA-FR-A69P-06	Melanoma	H1627Y	Missense	Medium
TCGA-HU-A4G8-01	Stomach	R2053Q	Missense	Medium
TCGA-HU-A4GQ-01	Stomach	A1289V3D	Missense	Medium
TCGA-HU-A4GQ-01	Stomach	A1377V3D	Missense	Medium
TCGA-HU-A4GQ-01	Stomach	E2697G3D	Missense	Medium
TCGA-IB-7651-01	Pancreas	N964H	Missense	Medium
TCGA-IG-A8O2-01	Esophagus	K1536Q	Missense	Medium
TCGA-LN-A4A3-01	Esophagus	E1804K	Missense	Medium
TCGA-LP-A4AV-01	Cervical	H1078Y	Missense	Medium

TCGA-QK-A8Z8-01	Head & neck	R1743H	Missense	Medium
TCGA-V1-A9O7-01	Prostate	W1772C	Missense	Medium
TCGA-33-4538-01	Lung squ	R459S	Missense	Neutral
TCGA-59-2354-01	Ovarian	S2896P	Missense	Neutral
TCGA-66-2754-01	Lung squ	E1127K	Missense	Neutral
TCGA-95-7039-01	Lung adeno	R2264K	Missense	Neutral
TCGA-A7-A13G-01	Breast	M2699K3D	Missense	Neutral
TCGA-A7-A4SC-01	Breast	T513S	Missense	Neutral
TCGA-A8-A0A6-01	Breast	V2432G	Missense	Neutral
TCGA-AA-3681-01	Colorectal	S2041F	Missense	Neutral
TCGA-AA-3977-01	Colorectal	Q1179K	Missense	Neutral
TCGA-AC-A23H-01	Breast	E2177K	Missense	Neutral
TCGA-AP-A051-01	Uterine	T2382M	Missense	Neutral
TCGA-AP-A056-01	Uterine	L2461F	Missense	Neutral
TCGA-AP-A059-01	Uterine	R2495S	Missense	Neutral
TCGA-AP-A059-01	Uterine	L98I	Missense	Neutral
TCGA-AP-A0LT-01	Uterine	T2382M	Missense	Neutral
TCGA-B5-A11E-01	Uterine	H134Y	Missense	Neutral
TCGA-B5-A11H-01	Uterine	E2844D	Missense	Neutral
TCGA-BL-A13I-01	Bladder	N2457Y	Missense	Neutral
TCGA-BR-4256-01	Stomach	T1227A	Missense	Neutral
TCGA-BR-8078-01	Stomach	A2785T	Missense	Neutral
TCGA-BR-A4QL-01	Stomach	R2491H	Missense	Neutral
TCGA-BS-A0UM-01	Uterine	A2137V	Missense	Neutral
TCGA-BT-A2LB-01	Bladder	K617E	Missense	Neutral
TCGA-C5-A1BQ-01	Cervical	V2615L3D	Missense	Neutral
TCGA-C8-A12T-01	Breast	D625H	Missense	Neutral
TCGA-CR-7380-01	Head & neck	N724K	Missense	Neutral
TCGA-CZ-5462-01	ccRCC	M888L3D	Missense	Neutral
TCGA-D1-A103-01	Uterine	A777T	Missense	Neutral
TCGA-D7-A6EY-01	Stomach	F765L	Missense	Neutral
TCGA-DA-A1IA-06	Melanoma	E1152K	Missense	Neutral
TCGA-DK-A3WW-01	Bladder	E2575Q3D	Missense	Neutral
TCGA-DS-A0VL-01	Cervical	T2196I	Missense	Neutral
TCGA-DX-A1KU-01	Sarcoma	N1240K	Missense	Neutral
TCGA-E2-A1IU-01	Breast	D2247Y	Missense	Neutral
TCGA-EB-A551-01	Melanoma	S782F	Missense	Neutral
TCGA-EE-A29V-06	Melanoma	P1066S	Missense	Neutral
TCGA-EE-A2GR-06	Melanoma	P588S	Missense	Neutral
TCGA-ET-A2MY-01	Thyroid	E741G	Missense	Neutral
TCGA-EX-A449-01	Cervical	R987Q	Missense	Neutral
TCGA-F7-A624-01	Head & neck	E2935K	Missense	Neutral
TCGA-FW-A3R5-06	Melanoma	L2970F	Missense	Neutral
TCGA-G3-A5SM-01	Liver	V1948M	Missense	Neutral
TCGA-GC-A3RC-01	Bladder	L1371V3D	Missense	Neutral
TCGA-GD-A3OP-01	Bladder	D1358N3D	Missense	Neutral
TCGA-H4-A2HQ-01	Bladder	R987Q	Missense	Neutral
TCGA-H7-A6C5-01	Head & neck	E2188Q	Missense	Neutral
TCGA-HC-8216-01	Prostate	V113L	Missense	Neutral
TCGA-IB-7651-01	Pancreas	A2372S	Missense	Neutral
TCGA-JY-A6FB-01	Esophagus	G717V	Missense	Neutral
TCGA-K4-A3WS-01	Bladder	R2303K	Missense	Neutral
TCGA-LP-A5U2-01	Cervical	E741K	Missense	Neutral
TCGA-OR-A5LJ-01	ACC	G1233S	Missense	Neutral
TCGA-SW-A7EA-01	Stomach	G2198V	Missense	Neutral
TCGA-UZ-A9PR-01	pRCC	E2991K	Missense	Neutral
TCGA-W5-AA39-01	Cholangiocarcinoma	A777T	Missense	Neutral
TCGA-X6-A8C3-01	Sarcoma	R274G	Missense	Neutral
TCGA-X6-A8C6-01	Sarcoma	P2036S	Missense	Neutral
TCGA-91-6847-01	Lung adeno	K813Sfs*9	FS del	
TCGA-BR-4361-01	Stomach	D2988Mfs*3	FS del	
TCGA-BR-8081-01	Stomach	D652Tfs*59	FS del	
TCGA-CG-4442-01	Stomach	D2988Mfs*3	FS del	
TCGA-CQ-7072-01	Head & neck	N1237Ifs*15	FS del	

TCGA-D3-A5GO-06	Melanoma	L1934Qfs*2	FS del
TCGA-HU-8602-01	Stomach	N2130Tfs*14	FS del
TCGA-HU-8602-01	Stomach	P178Lfs*33	FS del
TCGA-W5-AA2W-01	Cholangiocarcinoma	Y835Sfs*14	FS del
TCGA-A8-A097-01	Breast	D2988Gfs*2	FS ins
TCGA-CG-5721-01	Stomach	D2988Gfs*4	FS ins
TCGA-HC-A9TE-01	Prostate	N604Kfs*10	FS ins
TCGA-HU-A4H4-01	Stomach	L1020Ffs*33	FS ins
TCGA-N7-A4Y0-01	Uterine CS	D2988Gfs*2	FS ins
TCGA-RQ-AAAT-01	DLBC	I805Nfs*20	FS ins
TCGA-DQ-5629-01	Head & neck	A685_K686dup	IF ins
TCGA-G9-6373-01	Prostate	A685_K686dup	IF ins
TCGA-05-4420-01	Lung adeno	G2437L	Missense
TCGA-64-5774-01	Lung adeno	V2746L	Missense
TCGA-22-1016-01	Lung squ	E2238*	Nonsense
TCGA-2H-A9GR-01	Esophagus	Q412*	Nonsense
TCGA-44-7671-01	Lung adeno	K2635*3D	Nonsense
TCGA-AA-3949-01	Colorectal	S372*	Nonsense
TCGA-AG-A002-01	Colorectal	E628*	Nonsense
TCGA-AP-A051-01	Uterine	R1810*	Nonsense
TCGA-AX-A0J1-01	Uterine	W2332*	Nonsense
TCGA-B5-A0JY-01	Uterine	E1159*	Nonsense
TCGA-BR-A4QL-01	Stomach	R947*	Nonsense
TCGA-BS-A0UF-01	Uterine	E26*	Nonsense
TCGA-CV-A45W-01	Head & neck	E2853*	Nonsense
TCGA-D1-A17M-01	Uterine	R858*	Nonsense
TCGA-D8-A27G-01	Breast	Q61*	Nonsense
TCGA-DK-A1A3-01	Bladder	Q1281*3D	Nonsense
TCGA-ER-A19S-06	Melanoma	R1219*	Nonsense
TCGA-FW-A3R5-06	Melanoma	Q1214*	Nonsense
TCGA-IS-A3K6-01	Sarcoma	E1832*	Nonsense
TCGA-Q9-A6FU-01	Esophagus	E1223*	Nonsense
TCGA-VQ-A8P2-01	Stomach	E423*	Nonsense
TCGA-XJ-A9DI-01	Prostate	Q535*	Nonsense
TCGA-91-6829-01	Lung adeno	M1?	Nonstart
TCGA-CG-4306-01	Stomach	X1548_splice	Splice
TCGA-BR-8591-01	Stomach	L1685L	splice_region
TCGA-IB-7651-01	Pancreas	E871E	splice_region
