# Supplementary material for: Genotranscriptomic meta‐analysis of the CHD family chromatin remodelers in human cancers – initial evidence of an oncogenic role for CHD7
Source: Mol Oncol. 2017 Jul 21;11(10):1348–60. doi: 10.1002/1878-0261.12104 (PMC5623824; doi:10.1002/1878-0261.12104)
Supplement: Supplementary file 10 — Table S5. Frequency (%) of CHD genetic alterations and expression levels in five subtypes of TCGA breast cancers. [file MOL2-11-1348-s010.pdf]

Table S5. Frequency (%) of CHD genetic alterations and expression levels in five subtypes of TCGA breast cancers

	Gene	DNA alterations			mRNA Expression Levels		
		Amp/Gain	Deletion	Mutation	Z score>=1	1>Z score>-1	Z score<=-1
**Normal-Like**	CHD1	0.00	18.18	0.00	0.00	100.00	0.00
	CHD2	13.64	9.09	0.00	27.27	72.73	0.00
	CHD3	0.00	27.27	0.00	0.00	59.09	40.91
	CHD4	4.55	9.09	4.55	4.55	54.55	40.91
	CHD5	9.09	0.00	0.00	0.00	100.00	0.00
	CHD6	22.73	0.00	0.00	18.18	77.27	4.55
	CHD7	36.36	0.00	0.00	27.27	68.18	4.55
	CHD8	9.09	9.09	0.00	9.09	63.64	27.27
	CHD9	18.18	22.73	0.00	31.82	63.64	4.55
**Luminal A**	CHD1	22.96	7.16	0.49	12.10	87.65	0.25
	CHD2	12.84	15.31	0.49	16.05	74.81	9.14
	CHD3	4.94	51.36	1.73	8.15	69.38	22.47
	CHD4	14.57	11.11	2.22	21.23	66.67	12.10
	CHD5	1.48	31.36	1.98	0.25	99.75	0.00
	CHD6	34.57	2.72	2.47	26.91	69.88	3.21
	CHD7	42.22	3.95	1.98	20.49	67.16	12.35
	CHD8	11.60	11.36	1.48	22.47	68.64	8.89
	CHD9	11.85	64.20	1.48	12.59	79.75	7.65
**Luminal B**	CHD1	26.49	24.86	0.54	14.59	85.41	0.00
	CHD2	21.62	34.05	0.54	5.95	72.97	21.08
	CHD3	4.32	75.68	0.54	9.19	49.73	41.08
	CHD4	29.19	17.30	1.08	22.16	68.65	9.19
	CHD5	4.86	61.62	0.54	3.78	96.22	0.00
	CHD6	64.86	3.78	2.70	33.51	62.70	3.78
	CHD7	71.35	5.41	0.00	50.27	38.92	10.81
	CHD8	35.68	20.00	2.16	32.43	53.51	14.05
	CHD9	17.30	55.68	1.08	10.81	72.97	16.22
**HER2**	CHD1	12.12	40.91	0.00	1.52	98.48	0.00
	CHD2	15.15	39.39	1.52	4.55	69.70	25.76
	CHD3	1.52	86.36	3.03	1.52	34.85	63.64
	CHD4	22.73	25.76	3.03	21.21	59.09	19.70
	CHD5	10.61	50.00	1.52	7.58	92.42	0.00
	CHD6	48.48	7.58	1.52	24.24	69.70	6.06
	CHD7	57.58	9.09	3.03	40.91	53.03	6.06
	CHD8	27.27	19.70	1.52	21.21	65.15	13.64
	CHD9	25.76	42.42	3.03	6.06	75.76	18.18
**Basal-Like**	CHD1	0.77	80.00	0.77	0.77	97.69	1.54
	CHD2	28.46	40.77	1.54	10.00	57.69	32.31
	CHD3	8.46	67.69	2.31	2.31	40.00	57.69
	CHD4	53.08	20.00	2.31	24.62	57.69	17.69
	CHD5	24.62	41.54	1.54	2.31	97.69	0.00
	CHD6	44.62	20.00	0.77	5.38	54.62	40.00
	CHD7	69.23	10.00	0.77	71.54	25.38	3.08
	CHD8	14.62	60.00	0.77	3.08	59.23	37.69
	CHD9	16.92	42.31	2.31	6.15	68.46	25.38

Note: Amp/Gain= high-level amplification+low-level gain; Deletion = heterozygous deletion + homozygous deletion
