# Supplementary material for: Genotranscriptomic meta‐analysis of the CHD family chromatin remodelers in human cancers – initial evidence of an oncogenic role for CHD7
Source: Mol Oncol. 2017 Jul 21;11(10):1348–60. doi: 10.1002/1878-0261.12104 (PMC5623824; doi:10.1002/1878-0261.12104)
Supplement: Supplementary file 11 — Table S6. Frequency (%) of genetic and transcriptional alterations of CHDs in 1980 METABRIC breast cancers. [file MOL2-11-1348-s011.pdf]

Table S6. Frequency (%) of genetic and transcriptional alterations of CHDs in 1980 METABRIC breast cancers

GENE	Amp	Gain	Diploid	Hetloss	Homdel	Z Score >= 1	1 > Z Score > -1	Z Score <= -1
CHD1	0.051	3.788	92.374	3.737	0.051	15.15	69.29	15.56
CHD2	0.606	4.596	92.727	2.071	0.000	15.25	69.55	15.20
CHD3	0.051	0.657	83.636	15.556	0.101	13.69	72.98	13.33
CHD4	0.556	6.414	91.061	1.919	0.051	15.81	70.15	14.04
CHD5	0.000	1.667	91.717	6.566	0.051	6.16	88.54	5.30
CHD6	0.606	11.566	86.970	0.758	0.101	17.98	68.43	13.59
CHD7	2.576	23.182	72.828	1.313	0.101	24.24	61.21	14.55
CHD8	0.101	2.828	93.990	3.081	0.000	15.30	70.61	14.09
CHD9	0.505	3.081	71.919	24.141	0.354	14.49	67.58	17.93
