# Supplementary material for: Genotranscriptomic meta‐analysis of the CHD family chromatin remodelers in human cancers – initial evidence of an oncogenic role for CHD7
Source: Mol Oncol. 2017 Jul 21;11(10):1348–60. doi: 10.1002/1878-0261.12104 (PMC5623824; doi:10.1002/1878-0261.12104)
Supplement: Supplementary file 12 — Table S7. Frequency (%) of CHD genetic alterations and expression levels in five subtypes of Metabric breast cancers. [file MOL2-11-1348-s012.pdf]

Table S7. Frequency (%) of CHD genetic alterations and expression levels in five subtypes of Metabric breast cancers

	Gene	DNA alterations					mRNA Expression Levels		
		Amp	Gain	Diploid	Homdel	Hetloss	z score>=1	1>z score>-1	z score<=-1
**Normal-Like**	CHD1	0.00	1.51	97.49	1.01	0.00	9.05	72.86	18.09
	CHD2	1.01	2.01	94.97	2.01	0.00	10.05	79.40	10.55
	CHD3	0.00	1.01	90.45	8.54	0.00	18.59	69.35	12.06
	CHD4	0.00	1.51	97.49	1.01	0.00	8.54	74.87	16.58
	CHD5	0.00	1.01	96.98	2.01	0.00	3.02	92.46	4.52
	CHD6	0.00	5.53	93.97	0.50	0.00	8.54	74.37	17.09
	CHD7	2.01	10.55	86.43	0.50	0.50	10.55	75.88	13.57
	CHD8	0.00	1.51	95.98	2.51	0.00	13.57	78.39	8.04
	CHD9	1.01	0.50	91.46	7.04	0.00	24.62	68.34	7.04
**Luminal A**	CHD1	0.14	5.01	94.01	0.84	0.00	18.80	69.64	11.56
	CHD2	0.28	2.65	95.96	1.11	0.00	13.37	72.01	14.62
	CHD3	0.14	0.97	80.78	17.97	0.14	11.42	74.09	14.48
	CHD4	0.00	2.79	95.40	1.81	0.00	16.16	74.23	9.61
	CHD5	0.00	1.11	94.01	4.87	0.00	5.01	89.14	5.85
	CHD6	0.56	8.36	90.53	0.56	0.00	17.97	71.31	10.72
	CHD7	1.11	17.27	80.22	1.39	0.00	9.61	73.12	17.27
	CHD8	0.00	1.53	96.38	2.09	0.00	14.07	76.46	9.47
	CHD9	0.42	2.79	57.80	38.72	0.28	13.65	70.19	16.16
**Luminal B**	CHD1	0.00	5.74	91.80	2.25	0.20	24.80	68.03	7.17
	CHD2	1.23	4.92	91.60	2.25	0.00	18.65	67.21	14.14
	CHD3	0.00	0.41	74.18	25.20	0.20	13.11	74.59	12.30
	CHD4	0.20	5.33	90.57	3.69	0.20	11.68	72.13	16.19
	CHD5	0.00	1.84	85.66	12.30	0.20	5.74	87.50	6.76
	CHD6	1.43	21.72	75.82	0.82	0.20	29.51	62.30	8.20
	CHD7	4.71	34.43	59.22	1.64	0.00	25.00	54.71	20.29
	CHD8	0.20	4.30	92.21	3.28	0.00	13.52	69.06	17.42
	CHD9	0.20	3.89	63.52	31.76	0.61	9.22	66.39	24.39
**HER2**	CHD1	0.00	1.67	94.17	4.17	0.00	8.33	65.42	26.25
	CHD2	0.42	5.00	92.92	1.67	0.00	17.92	64.17	17.92
	CHD3	0.00	0.00	92.92	7.08	0.00	16.25	67.50	16.25
	CHD4	0.00	5.00	94.17	0.83	0.00	25.83	57.92	16.25
	CHD5	0.00	1.67	92.50	5.83	0.00	11.25	85.42	3.33
	CHD6	0.00	10.00	88.75	0.83	0.42	19.17	70.42	10.42
	CHD7	2.92	29.17	66.25	1.25	0.42	39.58	50.83	9.58
	CHD8	0.00	5.00	93.33	1.67	0.00	28.75	57.50	13.75
	CHD9	1.25	4.58	87.92	5.83	0.42	21.67	61.67	16.67
**Basal-Like**	CHD1	0.00	0.91	85.41	13.68	0.00	1.52	71.12	27.36
	CHD2	0.30	9.73	85.71	4.26	0.00	15.81	65.65	18.54
	CHD3	0.00	0.61	92.71	6.69	0.00	14.59	74.47	10.94
	CHD4	3.04	20.06	75.99	0.91	0.00	17.63	64.74	17.63
	CHD5	0.00	3.04	91.79	5.17	0.00	7.60	88.75	3.65
	CHD6	0.30	8.21	90.27	1.22	0.00	6.08	65.96	27.96
	CHD7	2.74	22.49	73.56	1.22	0.00	52.28	44.07	3.65
	CHD8	0.30	2.74	90.58	6.38	0.00	10.94	65.96	23.10
	CHD9	0.30	3.04	91.19	5.17	0.30	11.85	68.09	20.06
