# Supplementary material for: Genotranscriptomic meta‐analysis of the CHD family chromatin remodelers in human cancers – initial evidence of an oncogenic role for CHD7
Source: Mol Oncol. 2017 Jul 21;11(10):1348–60. doi: 10.1002/1878-0261.12104 (PMC5623824; doi:10.1002/1878-0261.12104)
Supplement: Supplementary file 13 — Table S8. Expression levels of CHD genes associated with NPI score in METABRIC breast cancer. [file MOL2-11-1348-s013.pdf]

Table S8. Expression levels of CHD genes associated with NPI score in METABRIC breast cancer

Gene	Low NPI Mean	High NPI Mean	*p* - value
**CHD1**	0.097	-0.034	7.08E-03
**CHD2**	0.022	0.018	9.33E-01
**CHD3**	-0.052	0.021	1.06E-01
**CHD4**	0.009	0.018	8.59E-01
**CHD5**	0.000	-0.018	7.13E-01
**CHD6**	0.205	0.038	9.10E-04
**CHD7**	-0.077	0.411	3.40E-20
**CHD8**	-0.004	0.007	8.18E-01
**CHD9**	0.021	-0.114	4.07E-03
