# Supplementary material for: Genotranscriptomic meta‐analysis of the CHD family chromatin remodelers in human cancers – initial evidence of an oncogenic role for CHD7
Source: Mol Oncol. 2017 Jul 21;11(10):1348–60. doi: 10.1002/1878-0261.12104 (PMC5623824; doi:10.1002/1878-0261.12104)
Supplement: Supplementary file 14 — Table S9. Correlation between mRNA expression of CHD7 and 46 candidate target genes in TCGA breast cancers. [file MOL2-11-1348-s014.pdf]

Table S9. Correlation between mRNA expression of CHD7 and 46 candidate target genes in TCGA breast cancers

Gene Symbol	Pearson Score	Spearman Score
CDK8	0.4	0.5
NRAS	0.07	0.36
SRC	0.23	0.32
SEC63	0.17	0.31
CORO1C	0.28	0.29
DYNC1LI2	0.25	0.29
CCNC	0.26	0.28
TULP3	0.28	0.28
EGFR	0.08	0.24
SOX11	0.24	0.24
CCSER2	0.16	0.19
FGD4	0.16	0.19
RBPJ	0.12	0.17
OXCT1	0.17	0.16
EEF1E1	0.14	0.14
SYNE2	0.16	0.14
SLC25A36	0.11	0.13
MYCN	0.1	0.12
LSM6	0.13	0.11
MOB3B	0.19	0.11
TADA1	0.18	0.1
PFKFB3	0.02	0.06
SPEG	0.16	0.06
RGS8	0	0.04
ITGA6	0.11	0.03
CPD	0.01	0.02
ZHX3	0.03	0.02
RAB33B	-0.02	-0.03
ABI2	0.02	-0.04
EIF1AY	-0.02	-0.05
FRMD4B	-0.09	-0.07
KLF15	-0.07	-0.08
TPM1	-0.04	-0.09
GRIA2	-0.09	-0.1
JAG1	-0.04	-0.1
TGFBR2	-0.08	-0.11
GLI2	-0.12	-0.12
BCAR3	-0.05	-0.15
CMTM3	-0.17	-0.15
KCTD17	-0.13	-0.16
CSRP1	-0.09	-0.17
HES5	0	-0.17
TMEM132C	-0.14	-0.18
MAPT	-0.13	-0.21
GLI3	-0.2	-0.25
ABAT	-0.19	-0.28
