# Supplementary material for: Genotranscriptomic meta‐analysis of the CHD family chromatin remodelers in human cancers – initial evidence of an oncogenic role for CHD7
Source: Mol Oncol. 2017 Jul 21;11(10):1348–60. doi: 10.1002/1878-0261.12104 (PMC5623824; doi:10.1002/1878-0261.12104)
Supplement: Supplementary file 15 [file MOL2-11-1348-s015.docx]

**Supplementary Figure Legends**

Figure S1. Expression levels of CHD7 across five subtypes of METABRIC breast cancer samples.

Figure S2. Kaplan-Meier plots of overall survival associated with mRNA expression levels of CHD7 in METABRIC breast cancers.

Figure S3. Expression levels of CHD7 based on RNA sequencing data from 78 breast cancer cell lines compared with four normal mammary epithelial cell lines. Cell lines: green indicates normal-like breast cell lines; light blue, Luminal A breast cancer cell lines; dark blue, Luminal B breast cancer cell lines; pink, HER2+ breast cancer cell lines; and red, basal-like breast cancer cell lines.

Figure S4. Expression levels of NRAS and MYCN, but not others, decreased in CHD7-knockdown SUM102 cells (* *p* < 0.05 and *** *p* < 0.001, Student’s *t*-test).

Figure S5. Phylogenetic analysis of chromodomain-containing proteins. The image was obtained

from the ChromoHub database (http://www.thesgc.org).
